# Molecular Phylogeny and Phylogeography of the Australian Freshwater Fish Genus *Galaxiella*, with an Emphasis on Dwarf Galaxias (*G. pusilla*)

**DOI:** 10.1371/journal.pone.0038433

**Published:** 2012-06-05

**Authors:** Peter J. Unmack, Justin C. Bagley, Mark Adams, Michael P. Hammer, Jerald B. Johnson

**Affiliations:** 1 National Evolutionary Synthesis Center, Durham, North Carolina, United States of America; 2 Evolutionary Ecology Laboratories, Department of Biology, Brigham Young University, Provo, Utah, United States of America; 3 Evolutionary Biology Unit, South Australian Museum, Adelaide, South Australia, Australia; 4 Australian Centre for Evolutionary Biology and Biodiversity, School of Earth and Environmental Science, University of Adelaide, Adelaide, South Australia, Australia; 5 Curator of Fishes, Museum and Art Gallery of the Northern Territory, Darwin, Northern Territory, Australia; 6 Monte L. Bean Life Science Museum, Brigham Young University, Provo, Utah, United States of America; Biodiversity Insitute of Ontario - University of Guelph, Canada

## Abstract

The freshwater fauna of Southern Australia is primarily restricted to the southwestern and southeastern corners of the continent, and is separated by a large, arid region that is inhospitable to this biota. This geographic phenomenon has attracted considerable interest from biogeographers looking to explain evolutionary diversification in this region. Here, we employed phylogenetic and phylogeographic approaches to evaluate the effect of this barrier on a group of four galaxiid fish species (*Galaxiella*) endemic to temperate Southern Australia. We also tested if continental shelf width has influenced connectivity among populations during low sea levels when rivers, now isolated, could have been connected. We addressed these questions by sampling each species across its range using multiple molecular markers (mitochondrial cytochrome *b* sequences, nuclear S7 intron sequences, and 49 allozyme loci). These data also allowed us to assess species boundaries, to refine phylogenetic affinities, and to estimate species ages. Interestingly, we found compelling evidence for cryptic species in *G. pusilla*, manifesting as allopatric eastern and western taxa. Our combined phylogeny and dating analysis point to an origin for the genus dating to the early Cenozoic, with three of the four species originating during the Oligocene-Miocene. Each *Galaxiella* species showed high levels of genetic divergences between all but the most proximate populations. Despite extensive drainage connections during recent low sea levels in southeastern Australia, populations of both species within *G. pusilla* maintained high levels of genetic structure. All populations experienced Late Pleistocene-Holocene population growth, possibly in response to the relaxation of arid conditions after the last glacial maximum. High levels of genetic divergence and the discovery of new cryptic species have important implications for the conservation of this already threatened group of freshwater species.

## Introduction

**Figure 1:**
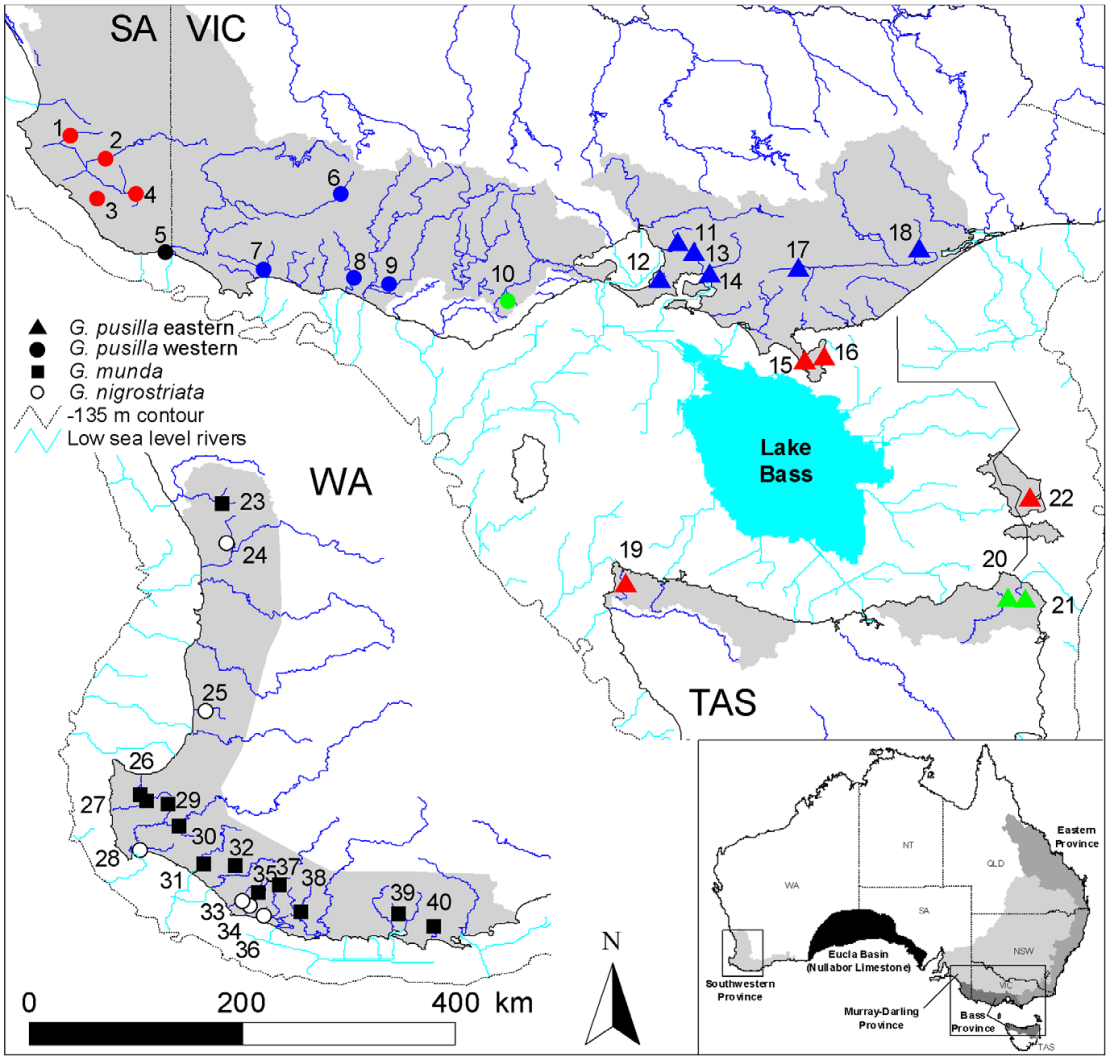
Localities for all *Galaxiella* samples examined. Refer to Table 1 for corresponding locality details. Each species is represented by a different symbol, with populations from both lineages of *G. pusilla* color coded to match Figs. 2, 3 and 5. Shaded areas refer to the general distribution of each *Galaxiella* species. Low sea level drainage patterns are shown to the minus 135 m bathymetric contour. Bathymetry predicts a large depression that we refer to as Lake Bass. The small inset of Australia shows the relevant biogeographic provinces, and the black central southern region represents the extent of the Eucla Basin.

Southern Australia provides an excellent geological setting for studying biogeographic patterns. Long-term aridity since the Oligocene created a vast desert region in southern Australia, isolating two moist temperate regions in the southwestern and southeastern parts of the continent. The biota that occupy these two regions have been isolated since at least Mid-Miocene (Fig. 1) [1], [2]. This has resulted in extreme endemism (∼75% of species) across most flora and fauna in southwestern Australia, an area recognized as one of 25 worldwide biodiversity hotspots [3]. In southeastern Australia, sea level changes have repeatedly connected and isolated Tasmania from mainland Australia, resulting in a vast, but temporary, increase in terrestrial habitat during low sea levels (over 83,000 km^2^; Fig. 1). This has resulted in a close relationship between the fauna of northern Tasmania and southern Victoria [4], [5]. Many studies have also examined the relationships of the biota across southern Australia [6]–[9]. In this study, we examine the effect of the aridification of southern Australia on the evolutionary diversification of a small group of fishes in the genus *Galaxiella* endemic to southern Australia.

Another earth history feature that could affect the distribution and genetic diversity of *Galaxiella* species is differences in the width of the continental shelf. Regions with narrow continental shelf should present limited opportunities for drainage connections during low sea levels, since few rivers are likely to connect (Fig. 1), whereas a broader continental shelf allows a greater area for rivers to traverse and potentially interconnect. Much of southern Australia has a relatively narrow continental shelf, thus most drainages remain isolated during low sea levels (Fig. 1). However, the region between northern Tasmania and southern Victoria has a broad continental shelf and most rivers in this area coalesce into a single large ‘Bass drainage’ which potentially results in high drainage connectivity during low sea levels (Fig. 1). In addition, during low sea levels a new north-south drainage divide emerges on the eastern edge of the Bass drainage (Fig. 1) which is broadly congruent with the biogeographic boundary between Bass and Eastern provinces [5]. Drainages to the east of that low sea level divide should experience long-term isolation, a pattern found in other freshwater fishes in this area [9], [10].

Freshwater fishes in the genus *Galaxiella* are widespread across southern Australia. As presently defined, the genus consists of three species of small, stout-bodied galaxiid fishes [11]. Two species, *G. nigrostriata* (blackstriped minnow) and *G. munda* (mud minnow), occur in southwestern Australia while a third species, *G. pusilla* (dwarf galaxias), occurs in southeastern Australia (Fig. 1). The three species differ only slightly in their morphology, with the primary differentiating characters relating to lateral striping and fin morphology [12], [13]. This has propagated confusion in southwestern Australia, where these species overlap in their distribution [13], [14]. Here, *G. munda* and *G. nigrostriata* occur in broad allopatry, but with limited local co-occurrence [13], [15]. Essentially, *G. nigrostriata* occur primarily closer to the coast in floodplain habitats that periodically desiccate, at which point in time they aestivate, whereas *G. munda* is usually found in smaller creeks and can occur further inland. Both species overlap to a limited extent in floodplain habitats that have seasonal connections to more permanent water bodies [16]. In contrast, *G. pusilla* spans the habitat types of both western species [17]–[19] and there is some evidence it may aestivate commensally with crayfish during desiccation [18]. All three species are small (up to 40–60 mm total length), have low fecundity (maximum of 250 eggs) and essentially live for only one year [19]–[21]. Importantly, all have experienced substantial range reductions, mainly due to habitat loss and introduced species such as *Gambusia holbrooki* (eastern mosquitofish). As a result, the two western species are considered ‘lower risk, near threatened’ by the Australian Society for Fish Biology (ASFB) [22], while *G. pusilla* is listed as ‘vulnerable’ by the ASFB, as well as in the International Union for Conservation of Nature Red List of Threatened Species [22], [23] and by the Australian federal government under the *Environment Protection and Biodiversity Conservation Act of 1999*.

Several studies have examined the taxonomy and relationships of *Galaxiella* species. The nomenclature of each *Galaxiella* species has remained largely stable since their description; however, their generic placement has changed. *Galaxiella pusilla* and *G. nigrostriata* were originally described as *Galaxias* species [24], [25]; however, both were subsequently merged by Scott [26] into the genus *Brachygalaxias*, from Chile [27]. Noting that Chilean *Brachygalaxias* were distinct from the Australian species, McDowall [28], [29] restricted *Brachygalaxias* to Chile, proposed *Galaxiella* for the Australian group and described *G. munda*. Several molecular and morphological studies have examined broader galaxiid relationships. Waters et al. [30] found *Galaxiella* species to be reciprocally monophyletic and most closely related to *Brachygalaxias* based on mitochondrial DNA sequences. A comprehensive morphological analysis also supported the monophyly of *Galaxiella* and *Brachygalaxias* [31]. Subsequent phylogenetic analyses based on 4.5 kb of mtDNA and nuclear DNA sequence data supported a close evolutionary relationship between *Galaxiella* and *Brachygalaxias*, but as sequential rather than as sister lineages, with both recovered as ancestral to the majority of other Galaxiidae species [32].

Molecular genetic variation within *Galaxiella* species from southwestern Australia has not been studied in detail. Smith et al. [33] examined allozyme variation in three populations of *G. nigrostriata* and found only one of 27 allozyme loci to be polymorphic. Phillips et al. [34] examined several populations of *G. munda* based on mtDNA control region and found 11 haplotypes from 92 individuals that grouped into four haplogroups, each differing by 4–7 bp. In southeastern Australia, Coleman et al. [35] examined 20 populations of *G. pusilla* using 11 microsatellite DNA loci and the mtDNA cytochrome oxidase I (COI) gene. Their major finding was of a deep east-west separation of *G. pusilla* into two lineages, consistent with the presence of two species. Coleman et al. [35] also reported moderate *F*_ST_ values and significant genetic structure among *G. pusilla* populations.

In order to determine the effect of earth history events on diversification in *Galaxiella*, a more integrative approach is needed; one that blends phylogeny and phylogeography. Although obviously relevant, previous single- and multi-locus phylogenetic analyses of *Galaxiella* used limited within-taxon sampling (one sample/species). Previous within-species comparisons in the two western *Galaxiella* species were also limited, as they addressed more specific local questions [33], [34]. For *G. pusilla*, Coleman et al. [35] employed good sampling with 10 individuals from each population but included only three populations outside of Victoria. Here, we improve on previous studies of the biogeography and population genetics of *Galaxiella* fishes. First, we use Gondwanan fragmentation and the well known geology of southern Australia to derive geologically-based calibration points for analyses and thereby estimate a time frame for diversification within the genus based on broader within-taxon sampling. This amounts to a ‘congeneric phylogeographical’ sampling approach. This has been shown to present a viable means of increasing systematic accuracy and improving historical inferences, by the recovery of polyphyly and ‘cryptic’ lineages, within vertebrate species [36], [37]. Second, we use a suite of molecular markers (mtDNA, nuclear DNA and allozymes where possible) to comprehensively assess species boundaries within all *Galaxiella* species based on more extensive geographic sampling. Third, we investigate phylogeographic patterns within each *Galaxiella* species. Fourth, we attempt to understand the effects of aridification on the evolutionary history of *Galaxiella* in southern Australia and the role that sea level changes had on the evolutionary histories within each *Galaxiella* species in southern Australia. Specifically, if shelf width is important then populations of *G. munda* and *G. nigrostriata* from southwestern Australia and populations of *G. pusilla* in the western portion of their range in southeastern Australia will have a high level of genetic divergence between drainages. In contrast, populations of *G. pusilla* that connect hydrologically to Bass drainage during low sea levels should have low genetic divergence, while populations to the east of Bass drainage should be distinct due to the low sea level drainage divide (Fig. 1). In addition, populations in the Bass drainage should show evidence of population expansion during the last glaciation due to the substantial increase in available habitat, whereas populations outside of this region should show little change as the amount of extra available habitat on the continental shelf was not substantially greater than today (Fig. 1).

## Materials and Methods

### Ethics Statement

Permission to undertake field work and collect specimens was obtained under the following permits: Victorian Fisheries research permit RP 581, Victorian Flora and Fauna permit 10002072, Victorian National Parks permit 10002154, Tasmanian Inland Fisheries Service permit 2003/12, Tasmanian Department of Primary Industries, Water and Environment permit TFA 03106, South Australian Primary Industries and Resources - Section 59 Exemption, Western Australian license to take fauna for scientific purposes SF006928. Specimens were obtained under Arizona State University Institutional Animal Care and Use Committee (IACUC) approval 09-1018R, Brigham Young University IACUC approval 070403 and University of Adelaide Animal Ethics Committee approval S-32-2002.

### Study Taxa and Sampling

The rarity and conservation status of these species limited our sample sizes. In many cases, there were only one or a few populations known per river basin. Our primary goal was to include one population per major drainage where each species is found. We examined samples of *Galaxiella* from 40 locations spanning virtually the entire distribution of this genus in Australia (Fig. 1 and Table 1). Samples from five of our six sampling locations for *G. nigrostriata* were provided by David Galeotti (Edith Cowan University). Fishes were collected with seine and dip nets and either frozen whole in liquid nitrogen or preserved in 95% ethanol in the field. Our sampling ranged from 1–11 individuals per locality, with most sites represented by 10 individuals within *G. pusilla* and 1–3 individuals in *G. munda* and *G. nigrostriata*, plus two sequences from GenBank for a total of 219 ingroup samples. Based on their close relationships to *Galaxiella* in previous molecular and morphological phylogenetic analyses [30]–[32], we also included five individuals from *Brachygalaxias bullocki* and *B. gothei* as outgroups in our DNA analyses (Table 1).

**Table 1:** Locality data for all individuals examined.

site no.	Locality	station code	species	cyt*b* N	S7 N	allozyme N
1	Bray Drain, Robe Naracoorte Rd, SA	FISH99	*G. pusilla*-west	9	1	9
2	Bakers Range, SA	FISH90	*G. pusilla*-west	11	1	11
3	Drain 32 outside Millicent, SA	FISH99	*G. pusilla*-west	9	1	10
4	Drain into Lake Letty (Dismal Swamp), SA	FISH90	*G. pusilla*-west	5	1	5
5	Swamp on entrance rd to Piccaninnie main pond, SA	FISH99	*G. pusilla*-west	10	1	10
6	Wannon R near Dunkeld, VIC	PU02-117	*G. pusilla*-west	10	1	10
7	Darlot Ck near Homerton, VIC	PU02-114	*G. pusilla*-west	10	1	10
8	Merri R, Grassmere, VIC	PU02-111	*G. pusilla*-west	6	1	6
9	Mount Emu Ck, Panmure, VIC	PU02-112	*G. pusilla*-west	2	1	2
10	Gosling Ck near Murroon, VIC	PU02-87	*G. pusilla*-west	9	1	9
11	Tirhatuan Swamp (now at LaTrobe University), VIC	PU03-03	*G. pusilla*-east	10	1	10
12	Tuerong Ck, VIC	PU03-04	*G. pusilla*-east	11		11
13	Cardinia Ck, Beaconsfield, VIC	PU02-102	*G. pusilla*-east	10	1	10
14	Yallock Ck, Koo Wee Rup, VIC	PU02-103	*G. pusilla*-east	10	1	10
15	Off Five Mile Track, Wilsons Prom, VIC	PU02-71	*G. pusilla*-east	9	1	10
16	Ck from Freshwater Lake, Wilsons Prom, VIC	PU02-70	*G. pusilla*-east	1	1	1
17	Moe R drains, Moe, VIC	PU02-80	*G. pusilla*-east	10	1	10
18	Perry R at Princess Hwy, VIC	PU02-68	*G. pusilla*-east	6	1	6
19	Swamp near Harcus, TAS	FISH98	*G. pusilla*-east	10	1	9
20	Gladstone Lagoon near Gladstone, TAS	FISH98	*G. pusilla*-east	2	1	2
21	Tributary to Icena Ck on rd to Ansons Bay, TAS	FISH98	*G. pusilla*-east	10	1	10
22	West of Five Hill Rd, Flinders Island, TAS	FISHY4	*G. pusilla*-east	11		11
23	Lennard Brook, WA		*G. munda*	1		
24	Melaleuca Park, Wetland EPP173, WA		*G. nigrostriata*	3	1	
25	Kemerton Nature Reserve, WA		*G. nigrostriata*	3	2	
26	Ironstone Gully, Buayanyup R, WA		*G. munda*	3	2	
27	Canebreak Pool, Margaret R, WA	PU09-58	*G. munda*	2		
28	Roadside pools off Scott R Rd, WA		*G. nigrostriata*	2	1	
29	Rosa Brook, Mowen Rd, WA	PU09-57	*G. munda*	2		
30	Milyeannup Brook, Brockman Hwy, Blackwood R, WA	PU09-53	*G. munda*	1		
31	Tributary of Donnelly R, upstream of Scott Rd, WA	PU09-52	*G. munda*	2	1	
32	Pemberton, WA	GenBank	*G. munda*	JN232599.1	JN232707.1	
33	Doggerup Ck, Windy Harbour Rd, WA	PU09-49	*G. nigrostriata*	2	1	
34	Chesapeake Rd west, WA		*G. nigrostriata*	2	2	
34	Chesapeake Rd west, WA	GenBank	*G. nigrostriata*	NC_008448.1		
35	Boorara Brook, Muirillup Rd, WA	PU09-48	*G. munda*	2	1	
36	Moores Hut Track, WA		*G. nigrostriata*	2	1	
37	Shannon R, South Western Hwy, WA	PU09-47	*G. munda*	2	1	
38	Deep R, Beardmore Rd below Fernhook Falls, WA	PU09-45	*G. munda*	2	1	
39a	Mitchell R, Denmark-Mt Barker Rd, WA	PU09-37	*G. munda*	2	1	
39b	Mitchell R, WA		*G. munda*	1		
40	Marbelup Brook, off Marbelup North Rd, WA	PU09-38	*G. munda*	2	1	
	Talka, Maule Basin, Chile	GenBank	*B. gothei*	JN232601.1	JN232709.1	
	Tributary to Laguna Saval, Valdivia Basin, Chile	GenBank	*B. bullocki*	JN232602.1	JN232710.1	
(LU)	8 km N of Los Ulmos, Valdivia Basin, Chile	DAN04-24	*B. bullocki*	1	1	
(RN)	Rio Negro, Maullin Basin, Chile		*B. bullocki*	2	2	

The site number refers to the location of each sample site as shown on Figure 1. The location column gives the general location of each sample. Station code refers to the field number or SAMA EBU code that samples are catalogued as. The last three columns provide the number of individuals (N) examined for each marker. Abbreviations: Ck, creek; Hwy, Highway; R, river; Rd, road; SA, Southern Australia; TAS, Tasmania; VIC, Victoria; WA, Western Australia.

### Sea Level Change Patterns

We employed a GIS approach to quantify several environmental factors needed to evaluate our hypotheses linked to changes in sea level. Datasets used to generate maps (e.g., Fig. 1) were obtained from the Digital Chart of the World [38] and manipulated in ArcInfo and ArcMap version 10 (Environmental Systems Research Institute, Redlands, CA). Bathymetric data were obtained from a 30 arc-second (ca. 1 km) dataset GEBCO 08 (September 2010 release, www.gebco.org) and manipulated to produce bathymetric contours and low sea level drainage patterns (Fig. 1) using the hydrological tools in ArcInfo.

### DNA Isolation, Amplification, and Sequencing

We extracted genomic DNA from muscle tissue for each specimen using DNeasy Tissue Kits (QIAGEN Inc., Chatsworth, CA). We amplified the mtDNA cytochrome *b* (cyt*b*) gene using two primers that flanked the gene. Most *G. pusilla* and *G. munda* samples were amplified using the primers Glu21 CCAGGACTAATGGCTTGAAAAA and GP.Thr27 TCTTCGGATTACAAAACCG. When this failed to produce sufficient PCR product, the gene was amplified in two halves using Glu21 - HD.GP GGRTTGTTTGAGCCTGTYTCGT and GP.505 TCTGTTCATAATGCAACCCT - GP.Thr27. For *G. nigrostriata* we also used the forward primers Glu18 TAACCAGGACTAATGRCTTGAA or Glu31 TGRCTTGAAAAACCACCGTTGT with HD.GP as well as the internal forward primer Sal.484 CAATGAATTTGAGGGGGRTTCTC or GN.484 CAATGAATTTGGGGTGGATT and the reverse primer GP.Thr27 or 15990 AGTTTAATTTAGAATCYTGGCTTTGG [39]. Several samples produced ambiguous chromatograms for the start of cyt*b*, as well as an unusual start to the gene (different start codon as well as deletion of the third codon and deletion of the last five bases of the Glu tRNA), hence, we amplified a 631 bp region from the middle of ND6 to base 345 of cyt*b* to confirm these sequences using the primers GalaND6F CTCTGGAAAAGGCTCCGCTG and Galacb346R CAAGAAGTAGTAATACAACACC. We also included the first two introns and the second exon of the nuclear S7 gene. All nuclear sequences were obtained by nested PCR using the following primers: 1F TGGCCTCTTCCTTGGCCGTC and 3R.24 AGCTGAGCCTTCAGGTCAGAG in the first reaction followed by 1F.2 CTCTTCCTTGGCCGTCGTTG and 3R GCCTTCAGGTCAGAGTTCAT in the second reaction. In a few cases, we had to use the internal primers 1F.2 and 2R.67 TACCTGGGARATTCCAGACTC, and 2F.2 GCCATGTTCAGTACCAGTGC plus 3R. Primers 1F and 3R are from Chow and Takeyama [40]. The first of these nested reactions were 25 µL. Final concentrations for polymerase chain reaction (PCR) components per 25 µL reaction were as follows: 25 ng template DNA, 0.25 µM of each primer, 0.625 units of Taq DNA polymerase, 0.1 mM of each dNTP, 2.5 µL of 10X reaction buffer and 2.5 mM MgCl_2_. Amplification parameters were as follows: 94°C for 3 min followed by 35 cycles of 94°C for 30 s, 48°C for 30 s, and 72°C for 90 s, and 72°C for 7 min. For the nested PCR our first reaction was 10 µL with the same PCR conditions listed above. This first PCR reaction was then diluted to 1∶99 and 1 µL from that was used in the second reaction. We examined PCR products on 1% agarose gels using SYBR safe DNA gel stain (Invitrogen, Eugene, OR, USA). We purified PCR products using a Montage PCR 96 plate (Millipore, Billerica, MA, USA). Sequences were also obtained via cycle sequencing with Big Dye 3.0 dye terminator ready reaction kits using 1/16th reaction size (Applied Biosystems, Foster City, CA). Sequencing reactions were run with an annealing temperature of 52°C following the ABI manufacturer’s protocol. We purified sequenced products using Sephadex™ columns (G.E. Healthcare, Piscataway, NJ). Sequences were obtained using an Applied Biosystems 3730 XL automated sequencer at the Brigham Young University DNA Sequencing Center. All haplotypes obtained in this study were deposited in GenBank, accession numbers JQ697745-JQ697836. Data files containing all individuals sequenced as well as various analysis files were deposited in Dryad, doi:10.5061/dryad.c3g8h.

### Analyses of DNA Sequence Data between *Galaxiella* Species

Sequences were edited using Chromas Lite 2.0 (Technelysium, Tewantin, Queensland, Australia) and imported into BioEdit 7.0.5.2 [41]. Cyt*b* was aligned by eye, while S7 sequences were aligned with the online version of MAFFT 6.822 [42] using the slow iterative refinement FFT-NS-i algorithm with the scoring matrix for nucleotide sequences set to 1PAM/K = 2, a gap opening penalty of 1.53 and an offset value of 0.1. All coding sequences were checked for unexpected frameshift errors or stop codons in Mega 5.05 [43]. Due to the presence of heterozygous individuals we phased the S7 data using the program Phase [44] as incorporated into DnaSP 5.10 [45]. The best-fitting model of molecular evolution for each gene (cyt*b*: GTR+I+G; S7: GTR+G) was estimated via AIC in ModelTest 3.7 [46] via PAUP* 4.0b10 [47]. We employed traditional tree-based maximum likelihood (ML) phylogenetic analyses using RAxML 7.2.8 [48], [49] with bootstrapping for 1000 pseudoreplicates, and the final ‘best’ ML tree was calculated using the GTRGAMMA model on the CIPRES cluster at the San Diego Supercomputer Center [50]. Trees were rooted with *Brachygalaxias bullocki* and *B. gothei*. The ML trees for cyt*b* and S7 were deposited in TreeBASE, accession number TB2:S12419 (http://purl.org/phylo/treebase/phylows/study/TB2:S12419). We calculated mean within- and among-taxon and population variation using p-distances in MEGA. We also reconstructed a haplotype network to resolve shallow relationships among closely related haplotypes [51]. We used TCS 1.21 [52] to create a network of the cyt*b* haplotypes using statistical parsimony with a 95% probability that no multiple substitutions had occurred. This failed to create a single network within both *G. munda* and *G. nigrostriata*, thus we reduced the probability to 92% and 93% respectively, at which point single networks were generated.

### Multi-locus Species Tree and Divergence Time Analysis

We used the Bayesian *BEAST algorithm [53] to reconstruct a species tree and to date clade divergences and coalescence times to the most recent common ancestor. We combined the data from cyt*b* and S7, included all individuals sequenced, then ran analyses using a relaxed molecular clock in BEAST 1.6.1 [54]. The full datasets for each gene were imported into BEAUti 1.7 (prerelease), which we used to generate the input file for BEAST. We incorporated two calibrations based on geological events. The first was the root of the tree, representing the split between *Brachygalaxias* and *Galaxiella*, as potentially congruent with the separation of Australia, Antarctica and South America. The terrestrial separation of Australia and Antarctica occurred at approximately 52 Ma [55], [56]. Second, the node between the southwestern and southeastern *Galaxiella* species was assigned a minimum age of 14 million years, representing the end of the Mid-Miocene transgression and formation of the Nullarbor Limestone over most of the Eucla Basin (Fig. 1) [1]. This node was defined as monophyletic based on our ML analyses and previous published work [30]–[32]. Three separate analyses were conducted using both calibration points in the same analysis, plus one analysis with each calibration used individually. Analyses were also conducted excluding the sequence data to check that posterior distributions were not heavily driven soley by our priors rather than the sequence data. We used an uncorrelated lognormal relaxed molecular clock based upon a lognormal prior using the ‘speciation birth death’ process. Both lognormal prior calibrations had a mean of 1.0, with standard deviations of 1.5 for the calibration of 52 Ma and 1.0 for the 14 Ma calibration. The best-fitting model of molecular evolution for each gene (cyt*b*: GTR+I+G; S7: GTR+G) was estimated via AIC in ModelTest. Analyses were run for 200 million generations, with parameters logged every 10,000 generations. Multiple runs were conducted to check for stationarity and that independent runs were converging on a similar result. Output from BEAST was examined in Tracer 1.5 with 10% burn-in and the tree results were summarized using TreeAnnotator 1.7 (prerelease).

### Analyses of Molecular Data within *G. pusilla*

We used multiple methods to examine within and between population genetic patterns in order to investigate the role of sea level changes in structuring genetic variation. We explored patterns of genetic variation in *G. pusilla* in greater depth as we had sampled a larger number of individuals with broader geographic coverage than the two southwestern *Galaxiella* species. Using DnaSP 5.10 [45], we estimated levels of genetic diversity, including numbers of haplotypes (*H*), haplotype diversity (*Hd*), and nucleotide diversity (π × 100), within each *G. pusilla* population and clade, and for all populations combined. To test the relative contribution of genetic variation to within and between population structure in *G. pusilla*, we performed an analysis of molecular variance (AMOVA) [57]. If genetic structuring is driven by isolation among drainages due to drainage basin boundaries, then we expect to see high levels of structuring among drainages. Cases where high amounts of variation can be explained between groups suggest an important historic barrier to gene flow exists coincident with the structure of the model. To evaluate movement between drainage basins, we combined populations into 15 groups based on drainage basins or close geographic proximity (i.e., the following populations were combined: 1–4, 11–12, 13–14, 15–16, 17–18; Fig.1). We ran these analyses with all populations included as well as limiting populations to only eastern and western *G. pusilla* lineages. AMOVA analyses were conducted in Arlequin 3.5 [58] with significance assessed using 10^4^ random permutations of the dataset.

### Historical Demography within *G. pusilla*

We further evaluated the evolutionary history of *Galaxiella* in southeastern Australia by testing hypotheses of population expansion within *G. pusilla* using several complementary analyses of our mtDNA data. First, we tested population expansion by using 10^4^ replicates of coalescent simulations in DnaSP to estimate Tajima’s *D* [59], Fu’s *F*_S_ [60], and *R*_2_ [61] and their upper and lower 95% confidence intervals (CIs) for local populations and all populations within each clade combined. Significant values of these statistics reveal deviations from neutral evolution and provide statistically ‘powerful’ tests of demographic-spatial population expansions [61]. Second, we used Bayesian skyline plots (BSPs) [62] to estimate past population dynamics through time within each *G. pusilla* clade. In pilot BEAST runs (ULN, MCMC = 10^6^) of cyt*b* variation for each clade, ucld.stdev parameter distributions clumped at zero, indicating highly clock-like data. We conducted BSP runs (MCMC = 2×10^8^; burn-in = 5×10^7^; piecewise-constant skyline model) in BEAST with strict clocks and evolutionary models from ModelTest (*G. pusilla* west: TrN+I+G; *G. pusilla* east: TrN+I+G). Taxon-specific rates of molecular evolution were unavailable, so we used conservative uniform priors spanning known mtDNA substitution rates ranging across teleost fish diversity (0.8–2.8% divergence/Myr [63]), allowing the program to estimate rates. BEAST runs partitioned sites by codon ((1+2), 3), unlinking parameters across positions and gave posterior node ages and their 95% highest posterior densities. Analyses excluding the DNA sequence data revealed resulting posterior distributions were not highly influenced by the priors. We calculated posterior distributions of *N*_e_ through time using TRACER 1.5. We used the Bayes factor method of Suchard et al. [64] implemented in TRACER to test the hypothesis that inferred BSPs fit the data better than constant models with similar priors. The best model had the highest marginal likelihood (± standard error; 1000 bootstrap pseudoreplicates) and was compared to the alternative using log_10_ Bayes factors (log_10_ *B*_10_). Log_10_ *B*_10_>2 units indicated definitive (very strong) evidence against the null model, while log_10_ *B*_10_<−2 units indicated very strong evidence for the null model [64], [65].

### Allozyme Analyses within *G. pusilla*

Since frozen tissues were available for virtually all *G. pusilla*, we were able to obtain comparative allozyme profiles for this species. These were used to independently assess the finding by Coleman et al. [35] of a major east-west dichotomy, and provide more appropriate molecular data for assessing whether any dichotomy reflected the presence of cryptic species. Three specimens of an outgroup species, *Galaxias olidus*, were also included in this analysis. We conducted allozyme electrophoresis of muscle homogenates on cellulose acetate gels (Cellogel™, Milan, Italy) following Richardson et al. [66]. Details of all enzyme and locus abbreviations, electrophoretic conditions, and stain recipes are contained in Richardson et al. [66] or Wallman and Adams [67], while the allozyme nomenclature follows Hammer et al. [68].

The genetic relationships among all sites were initially assessed by constructing a neighbour-joining (NJ) tree from a pairwise matrix of Nei’s [69] unbiased genetic distances (Nei *D*), following the methodology of Hammer et al. [10]. We then used GENEPOP 4.0 [70] to assess the raw allozyme genotypes for statistically-significant departures from Hardy-Weinberg expectations, for evidence of linkage disequilibrium at each individual site (for N>4), and to detect statistically significant differences in allele frequency among sites within each of the two major lineages evident within *G. pusilla*. Threshold probability values were adjusted for the use of multiple tests using sequential Bonferroni correction [71]. Finally, we employed the multivariate ordination technique Principal Coordinates Analysis (PCO) to visually assess the genetic affinities among all individuals within each lineage (details in [10]).

## Results

### Analyses of DNA Sequence Data between *Galaxiella* Species

Our molecular datasets provided clear discrimination among all described species. In addition, they revealed major and concordant genetic discontinuities consistent with cryptic speciation in *G. pusilla* as per Coleman et al. [35].

**Figure 2:**
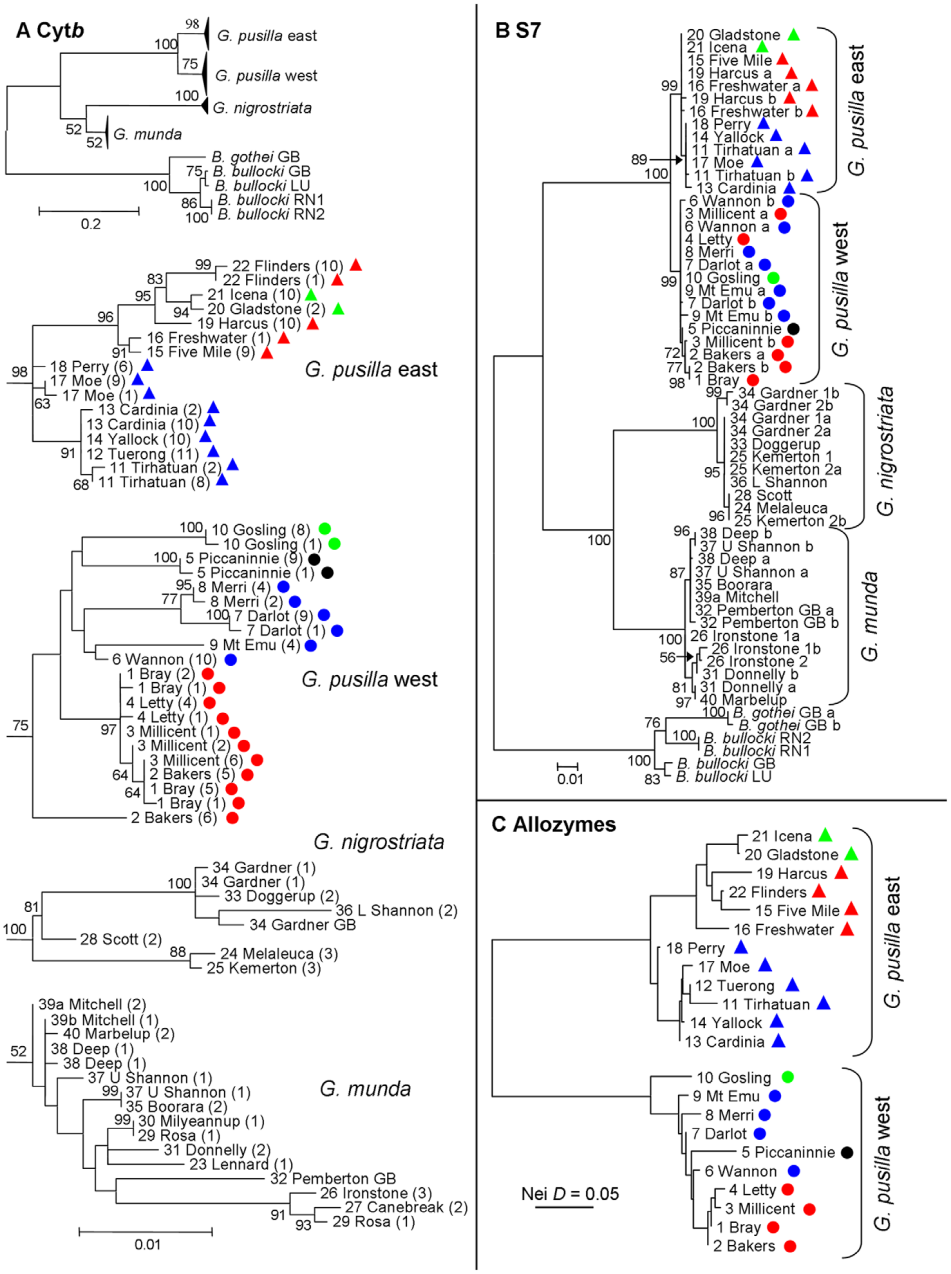
Phylogenetic results for all *Galaxiella* samples examined. Maximum likelihood trees for *Galaxiella* based on analysis of cytochrome *b* (A) and S7 (B) sequences and a neighbour joining tree for allozymes (C). Panel A shows the complete tree with operational taxonomic units (OTUs) for each *Galaxiella* species collapsed. Expanded trees within each species are shown below, with the lower scale bar applying to each of the four subtrees. Bootstrap values are based on 1000 pseudoreplicates. Trees are rooted with *Brachygalaxias*. Each OTU code is based on the sampling location number and name in Table 1 and Fig. 1, while the coloured symbols match Figs. 1, 3 and 5.

The cyt*b* dataset consisted of 1141 bp for 224 individual fish (Table 1). Removal of fish with identical haplotypes within populations reduced the dataset to 66 individuals. The number of individuals from a population with a given haplotype is provided after the population name in Fig. 2A. Based on this reduced dataset, 680 characters were invariant, 28 characters were variable but parsimony uninformative and 433 characters were parsimony informative. Both *G. munda* and *G. nigrostriata* had a deletion of their third cyt*b* codon, while the latter also had a premature stop codon in the third-to-last codon position (codon 378). Neither change is unusual for cyt*b* among fishes (P.J. Unmack, pers. obs.) or across various animal groups [72]. Furthermore, the complete mitochondrial genome (GenBank Accession No. NC_008448) closely matches our sequences (Fig. 2A) and was obtained via whole mtDNA amplification, which minimizes the chance of amplifying nuclear pseudogene copies (Masaki Miya, pers. comm.). Maximum likelihood recovered one tree for cyt*b* with a likelihood score of −5764.906494 (Fig. 2A). Mean p-distances for within- and among-taxon variation are presented in Table S1, while pairwise population comparisons for each species within *Galaxiella* are in Tables S2, S3, S4, S5.

The S7 dataset consisted of 899 bp for 43 individual fish (Table 1). Inclusion of individual alleles from 14 heterozygous individuals increased the dataset to 66 sequences. The number of heterozygous positions within an individual varied from one to three (all heterozygous individuals were phased into individual alleles prior to ML analyses). Based on this dataset, 535 characters were invariant, 10 characters were variable but parsimony uninformative and 354 characters were parsimony informative. Maximum likelihood recovered one tree for S7 with a likelihood score of −3378.210082 (Fig. 2B).

**Figure 3:**
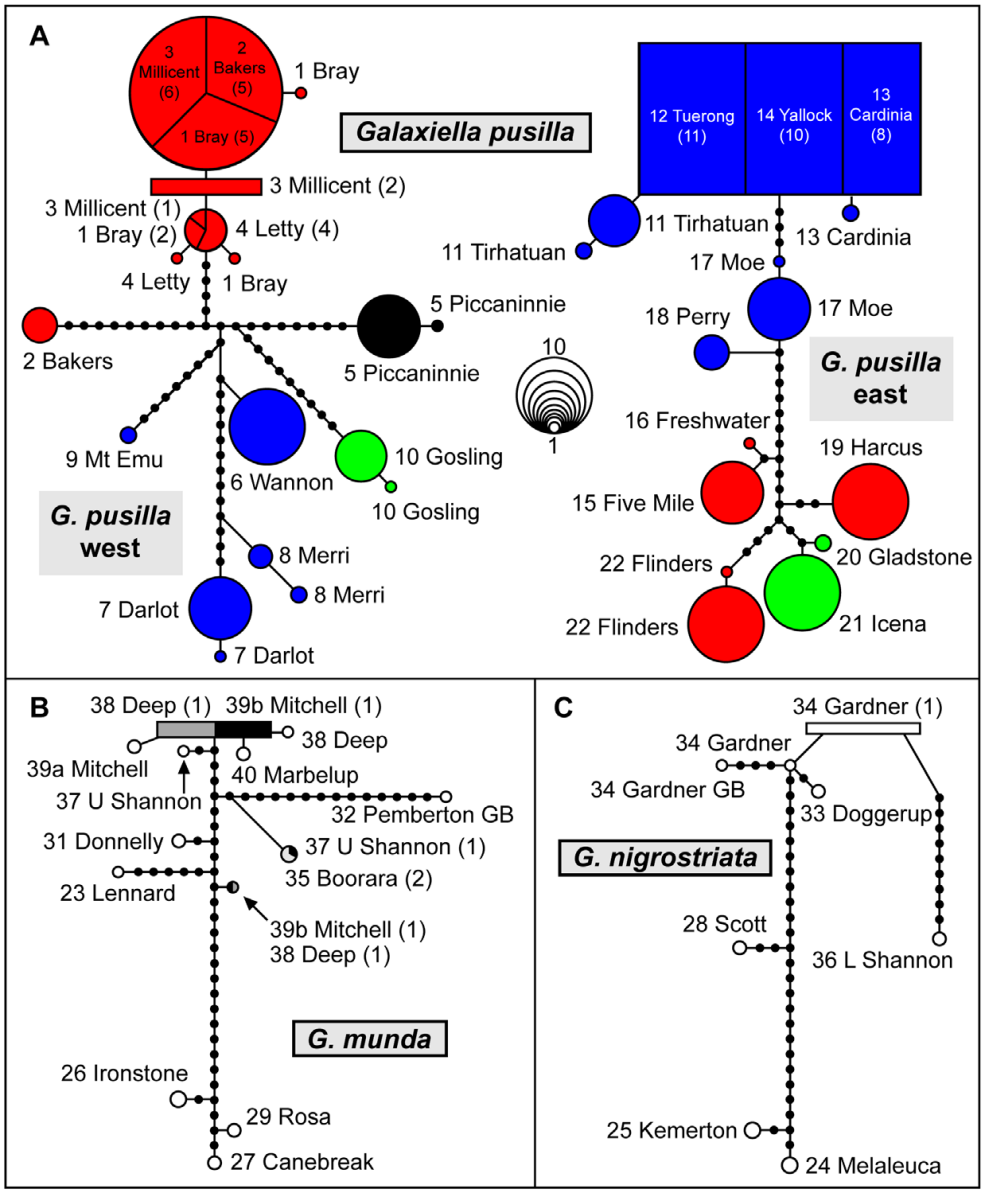
Haplotype networks for each *Galaxiella* species examined based on the cytochrome *b* gene. Each haplotype within *G. pusilla* (A) is color-coded relative to Figs. 1, 2 and 5. Circle size represents haplotype abundance; the key to circle size is in the center of panel A. The ancestral haplotype in each network is indicated by a box. Haplotype counts are given in parentheses when multiple populations share the same haplotype. Haplotype labels consist of the population number and name from Table 1 and Fig. 1. Unsampled haplotypes are represented by small filled circles.

The ML analyses of each gene recovered a nearly identical interspecific topology, with the only incongruence being among *Brachygalaxias* relationships. Cyt*b* placed *B. gothei* and *B. bullocki* as sister species, whereas S7 placed *B. gothei* in between the two *B. bullocki* lineages. Bootstrap support was high (>98%) for all deep nodes in the cyt*b* dataset, with the exception of the node uniting *G. munda* and *G. nigrostriata* (52%; Fig. 2). Our TCS networks provided a more detailed pattern of the relationships between cyt*b* haplotypes within each *Galaxiella* species, highlighting the large number of nucleotide differences between many populations (Fig. 3).

### Multi-locus Species Tree and Divergence Time Analysis

Species tree results produced the same topology as that recovered for the individual ML analyses of cyt*b* and S7 (Fig. 2), with Bayesian posterior probabilities equal to 1.0 for all internal nodes. Divergence dating results obtained using *BEAST are presented in Table 2. Running the analysis with the same settings, but without data confirmed that our input settings actually produced the desired prior probability distributions on our calibrated nodes and that our data were responsible for our results rather than our priors. Most statistics from all three analyses had ESS scores >400, demonstrating the chain was well sampled. Results were substantially different depending on the calibration used. When the single calibration of 14 Ma was used, all dates were much younger (Table 2). The combined calibration using 14 and 52 Ma and the single calibration of 52 Ma produced estimates twice as old, with the latter results being slightly higher than those of the combined calibration analysis (Table 2). The differences between these calibrations resulted in a doubling of the rates of evolution for both genes (Table 2).

**Table 2:** Results from BEAST dating analyses based upon different combinations of calibrations.

node	*Brachygalaxias*/*Galaxiella*	*Galaxiella*	*G. munda*/*G. nigrostriata*	*G. pusilla*-east/*G. pusilla*-west	*B. bullocki*/*B. gothei*	cyt*b*	S7
calibration	mean (95%)	mean (95%)	mean (95%)	mean (95%)	mean (95%)	mean	mean
52 and 14	54.4 (52.0–60.2)	30.9 (22.7–39.3)	20.8 (14.8–27.1)	5.2 (2.1–8.1)	9.6 (6.2–13.1)	0.835	0.197
52	55.8 (52.0–66.2)	34.3 (24.5–44.5)	22.5 (15.2–30.5)	5.7 (2.2–9.1)	10.3 (6.4–14.6)	0.777	0.182
14	26.8 (18.6–37.2)	16.7 (14.1–21.5)	10.9 (7.4–15.1)	2.7 (1.0–4.3)	5 (3.0–7.5)	1.604	0.375

The first line presents results based on both calibration points of 52 and 14 million years, while the second and third rows represent results under each individual calibration. The mean and 95% highest posterior densities are given for each node (in millions of years), and we report the per lineage mean rate of evolution per million years for each gene in the last two columns.

### Analyses of Molecular Data within *G. pusilla*

Estimates of genetic diversity based on cyt*b* (Table S6) revealed a pattern of relatively low genetic diversity within *G. pusilla* populations (mean *H* = 1.773; mean *Hd* ± one standard deviation (s.d.) = 0.212±0.090; mean π×100± s.d. = 0.059±0.014), but moderate genetic diversity within each clade (*G. pusilla* west: *H* = 23; *Hd* ± s.d. = 0.867±0.020; π×100± s.d. = 0.839±0.035; *G. pusilla* east: *H* = 16; *Hd* ± s.d. = 0.904±0.013; π × 100± s.d. = 1.189±0.048). In most cases, population genetic variation was characterized by 1 or 2 haplotypes per locality, with the second haplotype almost always being rare (1 or 2 individuals) and only three haplotypes shared across populations (Figs. 2, 3). Results from our AMOVA analyses found high levels of among-group structure consistent with the effects of strong isolation between populations. When all groups (N = 15) were compared, among-group comparisons accounted for 95.8% of the genetic variation. Within eastern *G. pusilla* (N = 8) and western *G. pusilla* (N = 7) among-group comparisons accounted for 90.2 and 72.8% of the variation respectively. All three among-group AMOVA values were statistically significant (P<0.05).

### Historical Demography within *G. pusilla*

**Figure 4:**
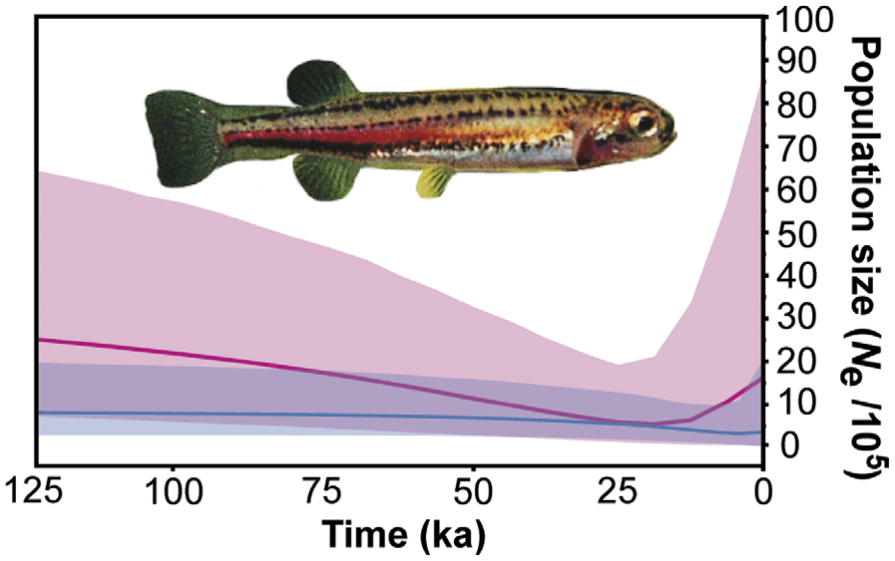
Historical demography of *Galaxiella pusilla* based on Bayesian skyline plots of female effective population size (*N*_e_) changes through time. Mean posterior *N*_e_ estimates for each species (darker lines) are bounded by upper and lower 95% highest posterior densities (*G. pusilla* west, pink shading; *G. pusilla* east, blue shading). The *x*-axis represents units of time in thousands of years ago (ka), scaled according to posterior mutation rates estimated in BEAST. The *y*-axis shows estimated population size in hundreds of thousands, calculated assuming a *G. pusilla* generation time equal to 1.0. The fish shown is a male *G. pusilla* west (by MPH).

Among the neutrality statistics computed for each *G. pusilla* population in Table S6, only *R*_2_ values were significant (mean *R*_2_ = 0.246, *P*<0.0001), supporting demographic-spatial expansion. Neutrality tests showed a similar pattern across *G. pusilla* clades, with only the *R*_2_ estimates being significant (*G. pusilla* west: *R*_2_ = 0.0916, *P*<0.0001; *G. pusilla* east: *R*_2_ = 0.0956, *P*<0.0001). Both Tajima’s *D* and Fu’s *F*_S_ have been shown to be conservative tests, especially with smaller sample sizes relative to *R*_2_ [61]. Bayesian skyline plots (with ESS scores >1000) revealed a gradual bottleneck in *G. pusilla* west female *N*_e_ since ∼125–50 ka, reaching its lowest point at 25 ka, followed by gradual recovery, then a sharp rise in the last ∼12 ka (Fig. 4). Eastern *G. pusilla* show a very gradual decline, then very slight Late Holocene (∼3 ka) rise in mean female *N*_e_. Comparing BSPs to constant demographic models supported BSP patterns. BSP models provided a better fit to the data within each *G. pusilla* clade (*G. pusilla* west: constant ln likelihood [*L*] ± standard error = −2038.14±0.10; BSP ln *L* = −2029.17±0.10; *G. pusilla* east: constant ln *L* = −1824.98±0.11; BSP ln *L* = −1819.78±0.11), and log_10_ Bayes factors provided very strong evidence against constant models (*G. pusilla* west: BSP log_10_B_10_ = 3.898; *G. pusilla* east: BSP log_10_ *B*_10_ = 2.261).

### Allozyme Analyses within *G. pusilla*

The allele frequencies at 49 putative allozyme loci for the 22 sites surveyed (representing 182 individuals; Table 1) are presented in Table S7. No site displayed any statistical evidence of either linkage disequilibrium or of genotype frequencies being incompatible with Hardy Weinberg expectations. These data unequivocally recovered the same two primary clades (Fig. 2C) as identified by both cyt*b* and S7 datasets. Eastern and western clades were diagnosable by fully-fixed differences at eight allozyme loci (*Acon1*, *Acp*, *Acyc*, *Dia*, *Est2*, *Ldh*, *Me1*, and *Sordh*; Table S7) and near-fixed differences (allowing a cumulative tolerance of 10% for shared alleles) at a further four loci (*Adh1*, *Fdp*, *Mpi*, and *PepA2*; Table S7). Additional genetic heterogeneity was also clearly evident among sites within clades, although in contrast to the cyt*b* data, the *G. pusilla* east clade displayed higher average levels of genetic divergence among sites (average Nei *D* = 0.078) than did the *G. pusilla* west clade (average Nei *D* = 0.044).

**Figure 5:**
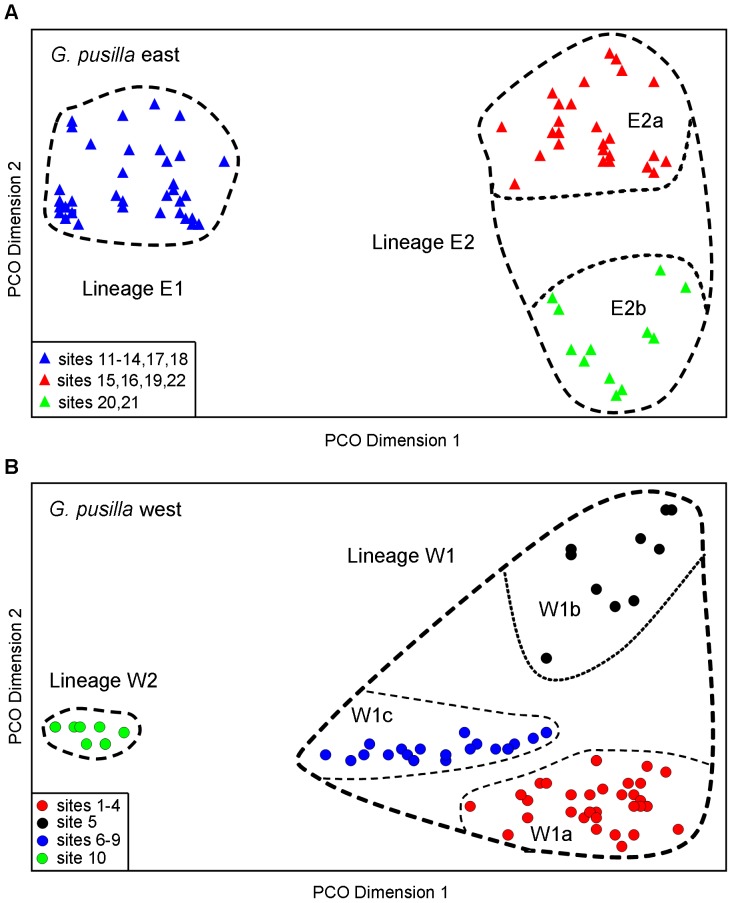
Principal coordinates analyses of *Galaxiella pusilla*. PCO for all 100 *G. pusilla* east (A) and all 82 *G. pusilla* west (B) individuals. Relative PCO scores are plotted for the first and second dimensions, which individually explain 41% and 10% (A) and 25% and 17% (B) respectively of the total multivariate variation present. Individuals are colour-coded relative to Figs. 1, 2 and 3.

Pairwise comparisons among sites within *G. pusilla* east revealed statistically significant differences in allele frequency, usually at multiple loci, in all but three instances (among sites 12, 13, and 14; Table S8). A PCO on all N = 100 individuals revealed the presence of two primary lineages (Fig. 5); lineage E1 comprised Victorian sites 11–18, while lineage E2 consisted of the two most southerly Victorian sites (15 and 16) plus all Tasmanian sites (sites 19–22). These two lineages were diagnosable by near-fixed differences at two loci (*Est1*, *Gsr*; Table S7), as well as by major differences in allele frequency (Δp>40%) at three other loci. Lineage E2 was further divisible into two sub-lineages E2a (sites 15, 16, 19, 22) and E2b (sites 20, 21), diagnosable by a fully-fixed difference at a single locus (*Acp*; Table S7) plus major differences in allele frequency at three other loci. Importantly, this phylogeographic structure is concordant with the cyt*b* haplotype network (Fig. 3A), and consistent with the S7 tree (which recovers lineages E1 and E2; Fig. 2B).

Pairwise comparisons among sites within *G. pusilla* west also revealed statistically significant differences in allele frequency, usually involving multiple loci, in all but four instances (sites 6 vs. 7, 1 vs. 2, 1 vs. 3, 2 vs. 4; Table S9). Two primary lineages, diagnosable by near-fixed differences at two loci (*PepA1*, *PepA2*; Table S7) plus major differences in allele frequency at five other loci, were evident in the PCO based on all N = 82 individuals (Fig. 5). Here lineage W2 consisted of the most easterly site (site 10), while lineage W1 comprised all other sites. Additional phylogeographic structure was detectable within the widespread lineage W1, although only one of the three sub-lineages (W1b = site 5) was allozymically well- differentiated from the other two sublineages (W1a = four western sites, W1c = four eastern sites) by major differences in allele frequency at multiple loci (Table S7). Nevertheless, this same phylogeographic arrangement of sites is also observable in the cyt*b* haplotype network (Fig. 3A).

## Discussion

### Species Relationships

Both single gene analyses (Fig. 2) and the combined (cyt*b* and S7) *BEAST Bayesian analysis revealed congruent topological relationships among *Galaxiella* species. These results were congruent with relationships shown by Burridge et al. [32] based on a single individual per taxon, although their dataset included two additional genes (RAG1 and 16 S) and morphological characters. Our results, showing a deep divergence between *G. pusilla* east and *G. pusilla* west using a suite of non-hypervariable nuclear genetic markers (and therefore more suitable for assessing species boundaries), were also consistent with the conclusions of Coleman et al. [35] based on mtDNA COI and nuclear microsatellites. We therefore refer to these lineages hereafter as distinct candidate species. Our results were strongly supported, with S7 and the combined analysis having high bootstrap values (100 and 1.0 respectively) for all between species relationships. High support from the cyt*b* analysis was found between *G. pusilla* species, however, bootstrap support was lower between *G. munda* and *G. nigrostriata*. Relationships between *Brachygalaxias* samples based on cyt*b* were congruent with recognised species, although *B. gothei* rendered *B. bullocki* paraphyletic in the S7 gene tree. Clearly, broader sampling within *B. bullocki* is required to clarify relationships.

### Timing of Divergences

Our divergence estimates varied greatly depending on whether we used the separation of Australian and South American species (min. age set to 52 Ma) or the east-west separation between *Galaxiella* species (min. age set to 14 Ma). Essentially, analyses using the older calibration point recovered mean ages slightly more than double those recovered using the younger calibration (Table 2). When both calibrations were included, estimates were up to ∼10% lower, especially on the upper 95% highest posterior distribution (HPD). While we present results from all three calibration combinations, we favour the results obtained using a single calibration of 52 Ma. The separation of southeastern and southwestern Australia by the formation of the Nullarbor Plain (Fig. 1) at 14 Ma and increasing aridity is only a minimum estimate and *Galaxiella* may have separated long before that event. Indeed, it has already been suggested that east-west separation of some groups predates this event by potentially tens of millions of years [8], [73]. This is despite the fact that many studies have found ages of separation to be fairly consistent with formation of the Nullarbor Plain [6]–[8], [74]. We are more confident that continental drift was responsible for their separation than some form of oceanic dispersal. Neither *Brachygalaxias* nor *Galaxiella* have any proclivity for marine environments today, nor do they possess any traits that imply a potentially diadromous life cycle. It is certainly not impossible that they were once diadromous, although this implies a substantial convergence in morphological and ecological characteristics, perhaps driven by adaptation to similar habitats. Our age estimates based on continental drift are similar to Burridge et al. [32]. While they did not calibrate their node for the split between *Galaxiella* and *Brachygalaxias*, they obtained a slightly older mean age than our analysis (58 vs. 55.8 Ma). However, their credibility interval was broader (45–72 vs. 52–66 Ma), probably due to their much larger dataset with multiple calibration points. The remaining divergences between each *Galaxiella* species estimated by Burridge et al. [32] were all younger than our results, with their mean ages usually being similar to our lowest 95% credibility interval values. In contrast, the age for both *Brachygalaxias* species was similar in both studies. It is also important to recognise that our estimate of 52 Ma for the separation of Australian and South American clades is only a minimum estimate. It is quite likely that the true divergence would have occurred earlier. This implies that all of our age estimates would be underestimated if the continental separation is an appropriate calibration point.

Divergence estimates between the three described *Galaxiella* species suggest that each represents an old lineage (even if our younger calibration analysis is considered; Table 2). We estimated the east-west separation of *Galaxiella* species across southern Australia at a mean age of 34.3 Ma (95% HPD of 24.5–44.5 Ma, Table 2). This mean age is older than that estimated for the separation of oldest extant pygmy perch species, the only other fishes with age estimates that share a distribution similar to *Galaxiella* [9]. At first glance this seems a slightly surprising result, as pygmy perches are often sympatric with all four *Galaxiella* species. However, the geographic distribution of pygmy perches extends into river basins that are more arid than those inhabited by *Galaxiella* species in southeastern and southwestern Australia [5], [15], [75]. Given that *Galaxiella* are adapted to exploit more ephemeral environments, they might be expected to persist under more arid conditions. However, increasing aridity would also increase the likelihood of extirpation, given *Galaxiella* species are more reliant on ephemeral habitats i.e., seasonally wet and dry on an annual basis. As aridity intensified across southern Australia [1], [2], these ephemeral habitats would be less reliably rewatered, thus eliminating populations through extended droughts.

Separation of *G. munda* and *G. nigrostriata* was estimated to have a mean age of 22.5 Ma (95% HPD of 15.2–30.5 Ma, Table 2). It is unclear which biogeographic scenarios might explain the evolution of the two southwestern *Galaxiella* species. Presumably populations were geographically separated at some stage and *G. nigrostriata* adapted to survive in temporary habitats. Alternatively, if aestivation was ancestral, then *G. munda* evolved the ability to compete and survive in permanent habitats. It seems likely that temporary habitats like those used by *G. nigrostriata* have a long history in southwestern Australia as they share this habitat with the ancient lineage of *Lepidogalaxias salamandroides* (salamanderfish) which also aestivates, has a similar distribution and is often sympatric with *G. nigrostriata* [15].

Separation of the two *G. pusilla* species had a mean age of 5.7 Ma (95% HPD of 2.2–9.1 Ma, Table 2). These species are allopatric, displaying no evidence for introgression, and their geographic separation seems to correspond to the western boundary of the low sea level Lake Bass drainage system (Fig. 1) [76], with populations from Mount Emu Creek/Hopkins River representing the western species. This contrasts strongly with patterns in other co-distributed fishes, where species-level separations or western range limits occur at the eastern side of the Lake Bass drainage [5]. Thus the scale of the genetic separation at this geographic location appears to be unique to *Galaxiella*, at least within freshwater fishes. No other fish species have divergences of this scale (i.e., species level differentiation) at this location, although within-species genetic breaks are present in several groups [9], [10], [68], [77], consistent with this boundary representing a barrier that is not frequently crossed by aquatic organisms. One major exception to the separation on the western edge of Lake Bass is population 10 from the Barwon River, which is thought to have become part of the Lake Bass drainage during low sea levels (Fig. 1). The close genetic affinity of population 10 to the western rather than the eastern *G. pusilla* species at first seems perplexing, as it runs counter to predictions from current drainage patterns. Based on the unique signature found at all molecular markers examined here (Figs. 2, 3, 5), plus those of Coleman et al. [35], it seems unlikely that population 10 represents a recent translocation. Instead, this outcome infers that the geomorphic history of the Barwon River basin differs somewhat from that of adjacent basins. While several authors [35], [78] have speculated on this issue in some detail relative to previously hypothesised high levels of Lake Corangamite [79], the lake expansion hypothesis needs to be re-examined geomorphically now that more accurate elevation and geological mapping is available. Clearly though, evidence does support some faunal exchange via Lake Corangamite.

### Phylogeographic Patterns within Species

Our most striking phylogeographic finding is a pattern of large genetic divergences between most populations within each of the two species of *G. pusilla* (Figs. 2, 3, 5 and Tables S2, S3). Results from cyt*b* and allozyme analyses were broadly congruent, although they differed in details. The extent of within-species genetic divergence differed between species as well as markers; for cyt*b*, eastern *G. pusilla* had an average within-group genetic divergence of 0.8%, while the western *G. pusilla* clade averaged 1.2% (Table S1). In contrast, allozyme results displayed an opposite pattern, with greater divergence among eastern *G. pusilla* populations than for the western species (Fig. 2C). AMOVA results for cyt*b* were more consistent with allozyme patterns, with more among-group variation explained in eastern *G. pusilla* populations relative to the western species (90.2% vs. 72.8% respectively). Five groups of populations had shared or closely related cyt*b* haplotypes (Figs. 2, 3 and Tables S2, S3) and these groups were broadly similar to those found for allozymes (Figs. 2, 5). Two of these groups of populations are found within drainages that are interconnected: populations 1–4 and 17–18 (Fig. 1). Three of these geographically proximate groups also had low average cyt*b* divergences (<0.2%): populations 11–14, 15–16 and 20–21 (Fig. 1); these were also similar based on allozyme variation (Fig. 5).

The broader relationships among eastern *G. pusilla* populations were slightly different between allozymes and cyt*b*. Allozyme analysis recovered populations clustering into three groups: (1) sites 11–14 and 17–18; (2) sites 15–16, 19, 22; and (3) sites 20–21 (Fig. 5). The main disparity between genetic markers was due to greater genetic divergence at cyt*b* between populations 11–14 relative to populations 17–18 and between populations 15–16 and those from Tasmania (Figs. 2, 3). Most relationships between western *G. pusilla* populations were similar between cyt*b* and allozymes. Both found that sites 1–4 had a close relationship and sites 7 and 8 were also similar at cyt*b* (p-distance of 0.5%); however allozymes grouped sites 6–9 together (Figs. 2, 3, 5). All other populations showed no particularly clear relationship to each other based on cyt*b*, and genetic divergences were higher (0.8–1.9%, Tables S2, S3). Allozyme analysis found that population 10 displayed 2–3 fixed differences from all other western *G. pusilla* populations, which only differed from each other based on allele frequencies. Coleman et al. [35] also found a similar pattern in their microsatellite analyses.

Against expectations, we found that neither inferred phylogenetic patterns, nor patterns of genetic structuring, corresponded very closely to predictions based on estimated low sea level drainage patterns. We predicted that populations 10–16, plus 19–20 would essentially show a low degree of genetic structure, since hydrological models predict that they would have been part of a larger continuous drainage system during historical periods of low sea level (Fig. 1). We expected populations 17–18 and 21–22 to be strongly genetically differentiated, as they are east of a hypothetical low sea level divide predicted from hydrology (Fig. 1). Lastly, populations 1–9 were expected to show relatively higher levels of genetic divergence. Although the latter pattern was indeed evident based on cyt*b* (but not for allozymes), neither of the other two predicted patterns were supported (Tables S2, S3). For example, haplotypes from sites 20 and 21, separated by the low sea level divide, only differed by a single mutation (Fig. 3A).

We predicted high connectivity between all eastern *G. pusilla* populations via the Bass Lake drainage, yet a complex population structure was evident, somewhat independent of drainage patterns (Figs. 1, 2, 3, 5). One extreme example is the relationship between sites 11–14 and 17–18, which are quite geographically distant. Based on low sea level drainage patterns, these populations should not have any lowland connections (Fig. 1), yet they are more closely related to each other than either is to two populations at an intermediate geographical distance (sites 15–16, Fig. 3). Thus, within interconnected drainages (i.e., sites 1–4, 17–18) or at small scales between some proximate populations (i.e., 11–14, 15–16, 20–21), *G. pusilla* shows evidence of recent genetic exchange based on the small proportion of genetic differences between them; or in a few cases, shared haplotypes. However, as soon as populations become more geographically distant, the levels of genetic divergence increase substantially. The fact that the low sea level drainage via Lake Bass has apparently not provided high population connectivity during the last glacial cycle is unexpected, as *G. pusilla* should have been almost continuously distributed throughout that region, given the potential connectivity when sea levels were low [76].

One possible caveat is that, during the last glacial maximum, conditions are thought to have been more arid than the present day [80], which may have limited population expansion across Lake Bass drainage. This aridity may have also limited floodplain connectivity between basins not connected via low sea level drainages, further restricting their potential to mix between basins. There is also evidence that Lake Bass may not have been fully fresh, or that salinity fluctuated due to decreased streamflow into the lake as a result of regional aridity [76], [81]. Additionally, some populations such as 15–16 may have resisted invasion due to competitive exclusion [82] and thus have retained distinctive haplotypes. Other possible explanations are that the patterns of low sea level drainages, based on bathymetry data, are not accurate or that other barriers existed that prevented gene flow. However, several species do show evidence for separation broadly consistent with this low sea level divide [9], [10]. That being said, the topographic difference (due east of population 16) across that low sea level drainage divide (Fig. 1) is very small and *G. pusilla* may have crossed it more readily than other species.

Results from historical demographic analyses provide an additional perspective on the history of *G. pusilla* populations. We predicted that *G. pusilla* should have experienced a large increase in population size as a vastly larger area of potential habitat (including Bass Lake) was created during periods of low sea level, but that populations beyond this region should not show evidence for population size expansion. Our results were not consistent with this hypothesis as we recovered genetic signatures of population expansion within all populations and clades irrespective of continental shelf width (Table S6). Although *R*_2_ statistics provided the only evidence for demographic-spatial population expansion (P<0.0001; Table S6) among the neutrality statistics we simulated, these statistics also likely had the most power (>60–80%) to reject the null hypothesis of demographic stasis (especially over Tajima’s *D*), given our small-moderate population sample sizes [61]. Model comparisons also supported Bayesian skyline models showing Late Pleistocene-Holocene increases in population size, although this estimated population expansion occurred either during or after sea levels had risen (Fig. 4). For instance, the approximate timing of the increase in population growth estimated from BSPs for *G. pusilla* east was very recent (∼3 ka), while estimates of increases in *G. pusilla* west population growth was slightly older (18–14 ka). These results may be more consistent with climatic changes since the last glacial maximum, primarily the amelioration of arid conditions [80], with earlier more intense aridity having reduced population connectivity. Nonetheless, given the time-dependency of molecular rates of evolution documented among freshwater fishes [83], and the notorious time lag between population divergence and concordant gene trees (i.e., coalescence [84]), it remains a possibility that the inferred timing of expansion within *G. pusilla* species is inaccurate. Based on the between-population patterns of genetic variation reported here, it seems intuitive that the growth/expansion of both *G. pusilla* species likely did not occur as a clean, unidirectional spatial expansion through drainage connections; rather, it may have corresponded to a period of wholesale average change in population size, hence the number of genetic mutations. Alternatively, the broad inferred changes in population dynamics within both species may reflect changes in the amount of between-population genetic structuring, rather than changes in population size through time.

*Galaxiella munda* and *G. nigrostriata* show even more extreme examples of population differentiation. Overall though, patterns were broadly similar to those in *G. pusilla*. Some proximate populations had low divergences (e.g., sites 32–33, 34 and 36, 38–39). Populations 24–25 were unusual in that they are separated by considerable distance, yet they were genetically quite similar (Fig. 3 and Table S5). Most other populations had high genetic divergences, typically from 0.7% to 3.0% (Tables S4, S5). Interestingly, despite being more common in elevations closer to sea level, *G. nigrostriata* displayed a higher degree of genetic differentiation than *G. munda* (Table S1).

### Conservation Implications

High levels of genetic divergence and the discovery of new cryptic species have important implications for the conservation of this threatened group of freshwater species. Molecular evidence suggests that *Galaxiella* represents an old lineage potentially dating back to the early Cenozoic (66–52 Ma), with three of its four species established by 44–15 Ma. All *Galaxiella* species typically have high levels of genetic divergences between all but the most proximate populations. Despite extensive drainage connections during recent low sea levels (∼18 ka) in southeastern Australia, populations in both species of *G. pusilla* maintain high levels of genetic structure. Broadly, the unequivocal evidence for cryptic species within *Galaxiella pusilla* s.l. heightens the need for conservation efforts to prevent species loss [85]. Genetic divergences within *Galaxiella* species imply that many populations are likely to contain unique combinations of genetic variation that will be important in planning for their long-term conservation and evolutionary potential [86].

## Supporting Information

Table S1 **Mean genetic divergences between species for cytochrome** ***b*** **calculated using p-distances.** The last column represents mean within species divergences.
(DOC)

Table S2 **Mean genetic divergences between western lineage populations of** ***Galaxiella pusilla*** **for cytochrome** ***b*** **calculated using p-distances.**
(DOC)

Table S3 **Mean genetic divergences between eastern lineage populations of** ***Galaxiella pusilla*** **for cytochrome** ***b*** **calculated using p-distances.**
(DOC)

Table S4 **Mean genetic divergences between populations of** ***Galaxiella munda*** **for cytochrome** ***b*** **calculated using p-distances.**
(DOC)

Table S5 **Mean genetic divergences between populations of** ***Galaxiella nigrostriata*** **for cytochrome** ***b*** **calculated using p-distances.**
(DOC)

Table S6 **Summary of genetic diversity of cytochrome** ***b*** **sequences sampled within** ***Galaxiella pusilla*** **populations and clades in this study.** Locality numbers, the number of individuals sampled, number of haplotypes (*H*), haplotype diversity (*Hd*) with its standard deviations, nucleotide diversity (π) with its standard deviations, and the results of coalescent simulations of three neutrality statistics, Tajima’s *D*, Fu’s *F*_S_, Ramos-Onsins and Rozas’ *R*_2_, are shown. Within-population values only represent genetically polymorphic sites; unlisted populations were monomorphic (*H* = 1; *Hd* = π = 0.000).
(DOC)

Table S7 **Allozyme frequencies at all variable loci for the 22 sites surveyed for** ***Galaxiella pusilla*** **.** Site codes follow Table 1. Frequencies of all but the rarer/rarest alleles are expressed as percentages and shown as superscripts (allowing the frequency of each rare allele to be calculated by subtraction from 100%). Maximum sample sizes per site are shown in brackets (asterisks indicate sample sizes of n = 3 for the designated sites and loci). A dash indicates insufficient enzyme activity at this locus. Invariant loci: Ald1*, Ald2*, Enol*, *Gapd1*, Glo*, *Gp*, *Gpi2*, Pgam*, and Pk*.
(DOC)

Table S8 **Summary of pairwise comparisons of allele frequency between sites for** ***Galaxiella pusilla*** **east.** Due to their small sample sizes, sites 16 and 20 were pooled with a geographically-proximate neighbour (sites 15 and 21 respectively). Lower triangle = number of loci displaying statistically-significant differences in allele frequency (P<0.05 after Bonferroni correction). Upper triangle = results of determining P-value for each population pair across all loci (Fisher’s method) after Bonferroni correction; *** = P<0.001, ** = P<0.01; ns = not significant.
(DOC)

Table S9 **Summary of pairwise comparisons of allele frequency between sites for** ***Galaxiella pusilla*** **west.** Site 9 was excluded from the analysis due to small sample size and lack of a geographically-proximate neighbour. Format as for Table S8.
(DOC)
