# Supplementary material for: Molecular Phylogeny and Phylogeography of the Australian Freshwater Fish Genus Galaxiella, with an Emphasis on Dwarf Galaxias (G. pusilla)
Source: PLoS One. 2012 Jun 5;7(6):e38433. doi: 10.1371/journal.pone.0038433 (PMC3367931; doi:10.1371/journal.pone.0038433)
Supplement: Table S1 — Mean genetic divergences between species for cytochrome b calculated using p-distances. The last column represents mean within species divergences. (DOC) [file pone.0038433.s001.doc]

Table S1. Mean genetic divergences between species for cytochrome *b* calculated using p-distances. The last column represents mean within species divergences.

| Species | No. | 1 | 2 | 3 | 4 | 5 | within |
| --- | --- | --- | --- | --- | --- | --- | --- |
| *G. pusilla* east | 1 |  |  |  |  |  | 0.8 |
| *G. pusilla* west | 2 | 9.1 |  |  |  |  | 1.2 |
| *G. munda* | 3 | 17.9 | 18.4 |  |  |  | 1.1 |
| *G. nigrostriata* | 4 | 22.4 | 21.6 | 15.4 |  |  | 1.6 |
| *B. bullocki* | 5 | 24.6 | 23.8 | 21.4 | 24.5 |  | 2.4 |
| *B. gothei* | 6 | 25.1 | 24.1 | 22.1 | 25.1 | 11.0 | n/c |
