# Supplementary material for: Molecular Phylogeny and Phylogeography of the Australian Freshwater Fish Genus Galaxiella, with an Emphasis on Dwarf Galaxias (G. pusilla)
Source: PLoS One. 2012 Jun 5;7(6):e38433. doi: 10.1371/journal.pone.0038433 (PMC3367931; doi:10.1371/journal.pone.0038433)
Supplement: Table S2 — Mean genetic divergences between western lineage populations of Galaxiella pusilla for cytochrome b calculated using p-distances. (DOC) [file pone.0038433.s002.doc]

Table S2. Mean genetic divergences between western lineage populations of *Galaxiella pusilla* for cytochrome *b* calculated using p-distances.

| Site | 1 | 2 | 3 | 4 | 5 | 6 | 7 | 8 | 9 |
| --- | --- | --- | --- | --- | --- | --- | --- | --- | --- |
| 1 Bray |  |  |  |  |  |  |  |  |  |
| 2 Bakers | 0.8 |  |  |  |  |  |  |  |  |
| 3 Millicent | 0.1 | 0.8 |  |  |  |  |  |  |  |
| 4 Letty | 0.2 | 0.8 | 0.2 |  |  |  |  |  |  |
| 5 Piccaninnie | 1.5 | 1.5 | 1.5 | 1.3 |  |  |  |  |  |
| 6 Wannon | 0.9 | 1.1 | 0.9 | 0.8 | 1.1 |  |  |  |  |
| 7 Darlot | 1.8 | 1.8 | 1.8 | 1.7 | 1.8 | 1.2 |  |  |  |
| 8 Merri | 1.6 | 1.6 | 1.6 | 1.4 | 1.5 | 1.0 | 0.5 |  |  |
| 9 Mt Emu | 1.3 | 1.6 | 1.3 | 1.3 | 1.6 | 0.9 | 1.8 | 1.7 |  |
| 10 Gosling | 1.5 | 1.6 | 1.5 | 1.5 | 1.6 | 1.2 | 1.8 | 1.9 | 1.8 |
