# Supplementary material for: Molecular Phylogeny and Phylogeography of the Australian Freshwater Fish Genus Galaxiella, with an Emphasis on Dwarf Galaxias (G. pusilla)
Source: PLoS One. 2012 Jun 5;7(6):e38433. doi: 10.1371/journal.pone.0038433 (PMC3367931; doi:10.1371/journal.pone.0038433)
Supplement: Table S3 — Mean genetic divergences between eastern lineage populations of Galaxiella pusilla for cytochrome b calculated using p-distances. (DOC) [file pone.0038433.s003.doc]

Table S3. Mean genetic divergences between eastern lineage populations of *Galaxiella pusilla* for cytochrome *b* calculated using p-distances.

| Site | 11 | 12 | 13 | 14 | 15 | 16 | 17 | 18 | 19 | 20 | 21 |
| --- | --- | --- | --- | --- | --- | --- | --- | --- | --- | --- | --- |
| 11 Tirhatuan |  |  |  |  |  |  |  |  |  |  |  |
| 12 Tuerong | 0.1 |  |  |  |  |  |  |  |  |  |  |
| 13 Cardinia | 0.1 | 0.0 |  |  |  |  |  |  |  |  |  |
| 14 Yallock | 0.1 | 0.0 | 0.0 |  |  |  |  |  |  |  |  |
| 15 Five Mile | 1.2 | 1.1 | 1.2 | 1.1 |  |  |  |  |  |  |  |
| 16 Freshwater | 1.2 | 1.1 | 1.2 | 1.1 | 0.2 |  |  |  |  |  |  |
| 17 Moe | 0.5 | 0.4 | 0.4 | 0.4 | 0.9 | 0.9 |  |  |  |  |  |
| 18 Perry | 0.5 | 0.4 | 0.5 | 0.4 | 0.9 | 0.9 | 0.2 |  |  |  |  |
| 19 Harcus | 1.6 | 1.5 | 1.5 | 1.5 | 0.7 | 0.7 | 1.2 | 1.2 |  |  |  |
| 20 Gladstone | 1.7 | 1.6 | 1.6 | 1.6 | 0.8 | 0.8 | 1.3 | 1.3 | 0.6 |  |  |
| 21 Icena | 1.5 | 1.4 | 1.4 | 1.4 | 0.8 | 0.8 | 1.1 | 1.1 | 0.6 | 0.2 |  |
| 22 Flinders | 1.6 | 1.6 | 1.6 | 1.6 | 1.0 | 1.0 | 1.2 | 1.3 | 0.8 | 0.7 | 0.7 |
