# Supplementary material for: Molecular Phylogeny and Phylogeography of the Australian Freshwater Fish Genus Galaxiella, with an Emphasis on Dwarf Galaxias (G. pusilla)
Source: PLoS One. 2012 Jun 5;7(6):e38433. doi: 10.1371/journal.pone.0038433 (PMC3367931; doi:10.1371/journal.pone.0038433)
Supplement: Table S4 — Mean genetic divergences between populations of Galaxiella munda for cytochrome b calculated using p-distances. (DOC) [file pone.0038433.s004.doc]

Table S4. Mean genetic divergences between populations of *Galaxiella munda* for cytochrome *b* calculated using p-distances.

| Site | 23 | 26 | 27 | 29 | 30 | 31 | 32 | 35 | 37 | 38 | 39a | 39b |
| --- | --- | --- | --- | --- | --- | --- | --- | --- | --- | --- | --- | --- |
| 23 Lennard |  |  |  |  |  |  |  |  |  |  |  |  |
| 26 Ironstone | 2.1 |  |  |  |  |  |  |  |  |  |  |  |
| 27 Canebreak | 2.1 | 0.5 |  |  |  |  |  |  |  |  |  |  |
| 29 Rosa | 1.4 | 1.0 | 1.0 |  |  |  |  |  |  |  |  |  |
| 30 Milyeannup | 0.7 | 1.6 | 1.8 | 0.8 |  |  |  |  |  |  |  |  |
| 31 Donnelly | 0.9 | 1.9 | 2.1 | 1.3 | 0.5 |  |  |  |  |  |  |  |
| 32 Pemberton | 1.6 | 2.3 | 2.5 | 1.9 | 1.4 | 1.6 |  |  |  |  |  |  |
| 35 Boorara | 1.0 | 1.7 | 1.8 | 1.2 | 0.6 | 0.5 | 1.3 |  |  |  |  |  |
| 37 U Shannon | 0.9 | 1.8 | 1.9 | 1.3 | 0.7 | 0.7 | 1.4 | 0.3 |  |  |  |  |
| 38 Deep | 0.8 | 1.9 | 2.1 | 1.3 | 0.7 | 0.7 | 1.6 | 0.6 | 0.4 |  |  |  |
| 39a Mitchell | 0.9 | 1.9 | 2.1 | 1.4 | 0.7 | 0.8 | 1.6 | 0.6 | 0.5 | 0.1 |  |  |
| 39b Mitchell | 0.8 | 1.8 | 2.0 | 1.3 | 0.6 | 0.7 | 1.5 | 0.5 | 0.4 | 0.0 | 0.1 |  |
| 40 Marbelup | 0.9 | 1.9 | 2.1 | 1.4 | 0.7 | 0.8 | 1.6 | 0.6 | 0.5 | 0.1 | 0.2 | 0.1 |
