# Supplementary material for: Molecular Phylogeny and Phylogeography of the Australian Freshwater Fish Genus Galaxiella, with an Emphasis on Dwarf Galaxias (G. pusilla)
Source: PLoS One. 2012 Jun 5;7(6):e38433. doi: 10.1371/journal.pone.0038433 (PMC3367931; doi:10.1371/journal.pone.0038433)
Supplement: Table S5 — Mean genetic divergences between populations of Galaxiella nigrostriata for cytochrome b calculated using p-distances. (DOC) [file pone.0038433.s005.doc]

Table S5. Mean genetic divergences between populations of *Galaxiella nigrostriata* for cytochrome *b* calculated using p-distances.

| Site | 24 | 25 | 28 | 33 | 34^GB^ | 34 |
| --- | --- | --- | --- | --- | --- | --- |
| 24 Melaleuca |  |  |  |  |  |  |
| 25 Kemerton | 0.3 |  |  |  |  |  |
| 28 Scott | 1.6 | 1.5 |  |  |  |  |
| 33 Doggerup | 2.5 | 2.5 | 1.5 |  |  |  |
| 34 Gardner GB | 2.6 | 2.5 | 1.6 | 0.5 |  |  |
| 34 Gardner | 2.3 | 2.3 | 1.4 | 0.2 | 0.4 |  |
| 36 L Shannon | 2.9 | 3.0 | 2.0 | 1.1 | 1.0 | 0.9 |
