# Supplementary material for: Molecular Phylogeny and Phylogeography of the Australian Freshwater Fish Genus Galaxiella, with an Emphasis on Dwarf Galaxias (G. pusilla)
Source: PLoS One. 2012 Jun 5;7(6):e38433. doi: 10.1371/journal.pone.0038433 (PMC3367931; doi:10.1371/journal.pone.0038433)
Supplement: Table S6 — Summary of genetic diversity of cytochrome b sequences sampled within Galaxiella pusilla populations and clades in this study. Locality numbers, the number of individuals sampled, number of haplotypes (H), haplotype diversity (Hd) with its standard deviations, nucleotide diversity (π) with its standard deviations, and the results of coalescent simulations of three neutrality statistics, Tajima’s D, Fu’s F S, Ramos-Onsins and Rozas’ R 2, are shown. Within-population values only represent genetically polymorphic sites; unlisted populations were monomorphic (H = 1; Hd = π = 0.000). (DOC) [file pone.0038433.s006.doc]

Table S6. Summary of genetic diversity of cytochrome *b* sequences sampled within *Galaxiella pusilla* populations and clades in this study. Locality numbers, the number of individuals sampled, number of haplotypes (*H*), haplotype diversity (*Hd*) with its standard deviations, nucleotide diversity (π) with its standard deviations, and the results of coalescent simulations of three neutrality statistics, Tajima’s *D*, Fu’s *F*_S_, Ramos-Onsins and Rozas’ *R*_2_, are shown. Within-population values only represent genetically polymorphic sites; unlisted populations were monomorphic (*H* = 1; *Hd* = π = 0.000).

| locality no. | N | *H* | *Hd* | s.d. *Hd* | π × 100 | s.d. π × 100 | *D* | *F*_S_ | *R*_2_ |
| --- | --- | --- | --- | --- | --- | --- | --- | --- | --- |
| 1 Bray | 9 | 4 | 0.694 | 0.147 | 0.127 | 0.032 | -0.0479ns | 0.251ns | 0.210*** |
| 2 Bakers | 11 | 2 | 0.545 | 0.072 | 0.813 | 0.108 | -0.0672ns | 0.249ns | 0.158*** |
| 3 Millicent | 9 | 3 | 0.556 | 0.165 | 0.063 | 0.022 | -0.0240ns | 0.287ns | 0.232*** |
| 4 Letty | 5 | 3 | 0.400 | 0.237 | 0.035 | 0.021 | -0.00407ns | 0.328ns | 0.331*** |
| 5 Piccaninnie | 10 | 3 | 0.200 | 0.154 | 0.018 | 0.014 | -0.00380ns | 0.325ns | 0.243*** |
| 7 Darlot | 10 | 2 | 0.200 | 0.154 | 0.018 | 0.014 | -0.00594ns | 0.312ns | 0.242*** |
| 8 Merri | 6 | 2 | 0.533 | 0.172 | 0.047 | 0.015 | -0.00438ns | 0.353ns | 0.295*** |
| 10 Gosling | 9 | 2 | 0.222 | 0.166 | 0.019 | 0.015 | -0.00493ns | 0.302ns | 0.255*** |
| 11 Tirhatuan | 10 | 2 | 0.356 | 0.159 | 0.031 | 0.014 | -0.00858ns | 0.287ns | 0.236*** |
| 13 Cardinia | 10 | 2 | 0.356 | 0.159 | 0.031 | 0.014 | -0.00268ns | 0.306ns | 0.236*** |
| 17 Moe | 10 | 2 | 0.200 | 0.154 | 0.018 | 0.014 | 0.00167ns | 0.316ns | 0.243*** |
| 22 Flinders | 11 | 2 | 0.182 | 0.144 | 0.016 | 0.013 | -0.00353ns | 0.292ns | 0.233*** |
|  |  |  |  |  |  |  |  |  |  |
| *G. pusilla* west | 100 | 23 | 0.867 | 0.0200 | 0.839 | 0.035 | -0.0778ns | -0.480ns | 0.0916*** |
| *G. pusilla* east | 81 | 16 | 0.904 | 0.0130 | 1.189 | 0.048 | -0.114ns | -0.513ns | 0.0956*** |

*** P<0.0001; ns, not significant (P>0.05); s.d., standard deviation.
