# Supplementary material for: Molecular Phylogeny and Phylogeography of the Australian Freshwater Fish Genus Galaxiella, with an Emphasis on Dwarf Galaxias (G. pusilla)
Source: PLoS One. 2012 Jun 5;7(6):e38433. doi: 10.1371/journal.pone.0038433 (PMC3367931; doi:10.1371/journal.pone.0038433)
Supplement: Table S7 — Allozyme frequencies at all variable loci for the 22 sites surveyed for Galaxiella pusilla . Site codes follow Table 1. Frequencies of all but the rarer/rarest alleles are expressed as percentages and shown as superscripts (allowing the frequency of each rare allele to be calculated by subtraction from 100%). Maximum sample sizes per site are shown in brackets (asterisks indicate sample sizes of n = 3 for the designated sites and loci). A dash indicates insufficient enzyme activity at this locus. Invariant loci: Ald1*, Ald2*, Enol*, Gapd1, Glo*, Gp, Gpi2, Pgam*, and Pk*. (DOC) [file pone.0038433.s007.doc]

Table S7. Allozyme frequencies at all variable loci for the 22 sites surveyed for *Galaxiella pusilla*. Site codes follow Table 1. Frequencies of all but the rarer/rarest alleles are expressed as percentages and shown as superscripts (allowing the frequency of each rare allele to be calculated by subtraction from 100%). Maximum sample sizes per site are shown in brackets (asterisks indicate sample sizes of n = 3 for the designated sites and loci). A dash indicates insufficient enzyme activity at this locus. Invariant loci: *Ald1**, *Ald2**, *Enol**, *Gapd1*, *Glo**, *Gp*, *Gpi2*, *Pgam**, and *Pk**.

|  | 1 | 2 | 3 | 4 | 5 | 6 | 7 | 8 | 9 | 10 | 11 | 12 | 13 | 14 | 15 | 16 | 17 | 18 | 19 | 20 | 21 | 22 |
| --- | --- | --- | --- | --- | --- | --- | --- | --- | --- | --- | --- | --- | --- | --- | --- | --- | --- | --- | --- | --- | --- | --- |
| Locus | (9*) | (11*) | (10*) | (5) | (10*) | (10*) | (10*) | (6*) | (2) | (9*) | (10*) | (11*) | (10*) | (10*) | (10*) | (1) | (10*) | (6*) | (9*) | (2) | (10*) | (11*) |
| *Acon1** | a | a^83^,b | a | a | a | a | a | b^83^,a | a^50^,b | b^67^,a | c | c | c | c | c | c | c | c | c | c | c | c |
| *Acon2* | b | b | b | b | b | b | b | b | b | b | b^95^,a | b | b | b | b | b | b | b | b | b | b^70^,c | b |
| *Acp* | a | a | a | a | a | a | a | a | a | a | b | b | b | b | b | b | b | b | b | c | c | b |
| *Acyc** | b | b | b | b | b^83^,a | b | b | b | b | b | c | c | c | c | c | c | c | c | c | c | c | c |
| *Ada* | d^50^,e | d^50^, | d^80^, | d^50^,e | d^75^,e | e^90^, | e^50^,d^30^ | d^80^, | d^50^, | e | d | d | d | d | c^90^,b | b | d | d^83^,e | d | d | b^55^,d | d |
|  |  | e^41^,b | b^10^,e |  |  | d^5^,b | b^15^,a | b^10^,e | b^25^,e |  |  |  |  |  |  |  |  |  |  |  |  |  |
| *Adh1* | d | d | d^95^,b | d | d | d^95^,c | d | d | d | e^67^,d | c | c^86^,a | c^90^,a | c^80^,a | c | c | c | c | c | c | c | c |
| *Adh2* | b | b | b | b | b | b | b | b | b | b | b | b | b | b | b | b | b^80^,a | b | b | b | b^90^,c | b |
| *Ca* | b^83^,a | b^95^,a | b | b^80^,a | b | b | b | b | b | b | b^65^,a | b^91^,a | b | b | a^95^,b | a | a^80^,b | b | a | a | a | a |
| *Ck* | b | b | b | b | b | b | b^90^,a | b | b | b | b | b | b | b | b | b | b | b | b | b | b | b |
| *Dia* | d | d^91^,e | d | d | d^95^,c | d | d^95^,e | d | d^50^,e | d | b | b | b^95^,c | b | b | b | b^80^,a | b | b | b | b | b^68^,a |
| *Est1* | b | b | b | b | b | b | b | b | b | b^94^,a | b | b | b | b | d^75^, | b | b | b | d | d | d | d |
|  |  |  |  |  |  |  |  |  |  |  |  |  |  |  | b^15^,c |  |  |  |  |  |  |  |
| *Est2* | e | e^73^,d | e^90^,d | d^80^,e | e | e | e | e | e | e | b | b | b^90^,a | b | b | b | b | b | b | b | b^70^,c | b |
| *Fdp* | a | a | a | a | c^75^,a | a | a | a | a | a | b^90^,d | d | d | d | c | c | d^85^,c | d | c^78^,d | d | d^70^,c | c |
| *Fum* | a | a | a | a | a^75^,b | a | a | a | a | a | a | a | a | a | a | a | a | a | a | a | a | a |
| *Gapd2* | b | b | b | b | b^85^,a | b | b | b | b | b | b | b | b | b | b | b | b | b | b | b | b | b |
| *Got1* | a | a | a | a | a | a | a | a | a | a | a | a | a | a | a | a | a | a^67^,b | a | a | a | a^95^,b |
| *Got2* | c | c^91^,b | c | c^90^,a | c | c | c | c | c | c | c | c | c | c | c | c | c | c | c | c | c | c |
| *Gpi1* | b | b | b | b^90^,a | a^50^,b | b | b | b | b^75^,c | b | b | b | b | b | b | b | b | b | b | b | b | b |
| *Gsr* | c | c | c | c | c | c | c | c | c | c | c^95^,e | c | c^85^,d | c | b | b | c | c | a^61^,b | b | b | b^64^,c |
| *Idh1** | b | b | b | b | b | b | b | b | b | b | a^50^,b | a^67^,b | b^83^,a | b | b | b | b | b | b | b | b | b |
| *Idh2** | b | b | b | b | b | b | b | b | a^75^,b | b | b | b | b | b | b | b | b | b | b | b | b | b |
| *Ldh* | a | a | a | a | a | a | a | a | a | a | b | b | b | b | b | b | b | b | b | b | b | b |
| *Mdh1* | a^67^,c | a^73^,c | a^85^,c | a^80^,c | c | c | c | c | c | c | c | c | c | c | c | c | c | c | c | c | b^50^,c | c |
| *Mdh2* | b | b^95^,c | b | b | a^85^,b | b | b | b | b | b | b | b | b | b | b | b | b | b | b | b | b | b |
| *Me1** | a | a | a | a | a | a | a | a | a | a | b^83^,c | b | b | b | b^50^,c | b | b | b | b | b | b | c^67^,b |
| *Me2* | b^67^,d | b^82^,d | b^80^,d | b^50^,d | b^60^, | b | b | b | b | b | b^75^,a | b^68^,a | b^80^,a | b^70^,a | b^65^,a | b | b^70^,a | b^75^,a | b^78^,a | b^75^,a | b^65^,a | b^59^,a |
|  |  |  |  |  | c^35^,d |  |  |  |  |  |  |  |  |  |  |  |  |  |  |  |  |  |
| *Mpi* | d | d | d | d^90^,e | d^95^,b | d | d | d | d | d | a^65^,c | c^82^,a | c^95^,a | c | c | c | c | c | c | c | c | c^77^,b |
| *Ndpk** | a | a | a | a^90^,b | a | a | a^83^,b | a | a | a | a | a | a | a | a | a | a | a | a | a | a | a |
| *Np* | b | b | b | b | b | b | b | b | b | b | a^60^,b | b | b | b | b | b | b | b | b | b | b | b |
| *PepA1* | b | b | b | b | b | b | b | b | b^75^,a | a | b | b | b | b | b | b | b | b | b | b | b | b |
| *PepA2* | c^94^,d | c^95^,d | c^95^,d | c^90^,d | c^90^,a | c | c^95^,a | c | a^50^,c | f^34^, | d | d | d | d | d | d | d^85^,e | d | c^67^,d | d | d | d |
|  |  |  |  |  |  |  |  |  |  | d^33^,b |  |  |  |  |  |  |  |  |  |  |  |  |
| *PepB* | b | b | b | b | b | b | b | b | b | b | a | a | a^85^,b | a | b | b | a | b | b | b | b | b |
| *PepD* | a | a | a | a | a | a^90^,c | a^55^, | a | a^75^,c | c^94^,a | c | c | c^95^,b | c | b^65^,c | c | c | c | c | c | c | c |
|  |  |  |  |  |  |  | c^30^,d |  |  |  |  |  |  |  |  |  |  |  |  |  |  |  |
| *6Pgd* | d | d^95^,e | d | d | d^60^, | d | d | d | d | d^61^,b | d | d | d | d | d | d | d | d | d^89^,a | d | d | d |
|  |  |  |  |  | e^35^,c |  |  |  |  |  |  |  |  |  |  |  |  |  |  |  |  |  |
| *Pgk** | b | b^83^,a | b | b | b | b | b | b | b | b | b | b | b | b | b | b | b | b | b | b | b | b |
| *Pgm* | c^71^,d^1^ | c^77^,b | c^35^, | c^40^, | d^70^,c | c^85^,a | c^75^, | e^67^,c | c^75^,b | c | c | c | c | c | c | c | c | c | c | c | c | c |
|  | ^7^,a^6^,b |  | d^30^, | d^40^,b |  |  | a^20^,e |  |  |  |  |  |  |  |  |  |  |  |  |  |  |  |
|  |  |  | b^20^,a |  |  |  |  |  |  |  |  |  |  |  |  |  |  |  |  |  |  |  |
| *Sordh** | a | a | a | a | a | a | a | a | a | a | b | b | b | b | b | b | b | b | b | b | b | b |
| *Tpi1* | b | b | b^90^,a | b | b | b | b | b | b^50^,c | b | b | b | b | b | b | b | b | b | b | b | b | b |
| *Tpi2** | a | a | a | a | a | a | a | a | a | a | - | - | - | - | - | - | - |  | a | a | a | a |
| *Ugpp** | a | a | a | a^90^,b | a | a | a | a | a | a | a | a | a | a | a | a | a | a | a | a | a | a |
