# Supplementary material for: Molecular Phylogeny and Phylogeography of the Australian Freshwater Fish Genus Galaxiella, with an Emphasis on Dwarf Galaxias (G. pusilla)
Source: PLoS One. 2012 Jun 5;7(6):e38433. doi: 10.1371/journal.pone.0038433 (PMC3367931; doi:10.1371/journal.pone.0038433)
Supplement: Table S8 — Summary of pairwise comparisons of allele frequency between sites for Galaxiella pusilla east. Due to their small sample sizes, sites 16 and 20 were pooled with a geographically-proximate neighbour (sites 15 and 21 respectively). Lower triangle = number of loci displaying statistically-significant differences in allele frequency (P<0.05 after Bonferroni correction). Upper triangle = results of determining P-value for each population pair across all loci (Fisher’s method) after Bonferroni correction; *** = P<0.001, ** = P<0.01; ns = not significant. (DOC) [file pone.0038433.s008.doc]

Table S8. Summary of pairwise comparisons of allele frequency between sites for *Galaxiella pusilla* east. Due to their small sample sizes, sites 16 and 20 were pooled with a geographically-proximate neighbour (sites 15 and 21 respectively). Lower triangle = number of loci displaying statistically-significant differences in allele frequency (P < 0.05 after Bonferroni correction). Upper triangle = results of determining P-value for each population pair across all loci (Fisher's method) after Bonferroni correction; *** = P < 0.001, ** = P < 0.01; ns = not significant.

| Site | 21+20 | 19 | 22 | 18 | 17 | 16+15 | 14 | 13 | 12 | 11 |
| --- | --- | --- | --- | --- | --- | --- | --- | --- | --- | --- |
| 21+20 |  | *** | *** | *** | *** | *** | *** | *** | *** | *** |
| 19 | 7 |  | *** | *** | *** | *** | *** | *** | *** | *** |
| 22 | 5 | 3 |  | *** | *** | *** | *** | *** | *** | *** |
| 18 | 6 | 6 | 4 |  | *** | *** | *** | *** | *** | *** |
| 17 | 7 | 6 | 4 | 2 |  | *** | *** | *** | ** | *** |
| 16+15 | 6 | 6 | 4 | 6 | 7 |  | *** | *** | *** | *** |
| 14 | 7 | 7 | 5 | 1 | 1 | 7 |  | ns | ns | *** |
| 13 | 7 | 7 | 5 | 1 | 1 | 7 | 0 |  | ns | *** |
| 12 | 7 | 7 | 5 | 1 | 2 | 7 | 0 | 0 |  | *** |
| 11 | 10 | 9 | 7 | 4 | 3 | 9 | 3 | 3 | 2 |  |
