# Supplementary material for: Molecular Phylogeny and Phylogeography of the Australian Freshwater Fish Genus Galaxiella, with an Emphasis on Dwarf Galaxias (G. pusilla)
Source: PLoS One. 2012 Jun 5;7(6):e38433. doi: 10.1371/journal.pone.0038433 (PMC3367931; doi:10.1371/journal.pone.0038433)
Supplement: Table S9 — Summary of pairwise comparisons of allele frequency between sites for Galaxiella pusilla west. Site 9 was excluded from the analysis due to small sample size and lack of a geographically-proximate neighbour. Format as for Table S8. (DOC) [file pone.0038433.s009.doc]

Table S9. Summary of pairwise comparisons of allele frequency between sites for *Galaxiella pusilla* west. Site 9 was excluded from the analysis due to small sample size and lack of a geographically-proximate neighbour. Format as for Table S8.

| Site | 10 | 8 | 7 | 6 | 5 | 4 | 3 | 2 | 1 |
| --- | --- | --- | --- | --- | --- | --- | --- | --- | --- |
| 10 |  | *** | *** | *** | *** | *** | *** | *** | *** |
| 8 | 6 |  | ns | *** | *** | *** | *** | ** | *** |
| 7 | 6 | 1 |  | ns | *** | *** | *** | *** | *** |
| 6 | 5 | 2 | 0 |  | *** | *** | *** | ** | *** |
| 5 | 10 | 4 | 6 | 6 |  | *** | *** | *** | *** |
| 4 | 7 | 3 | 3 | 3 | 4 |  | *** | ns | ns |
| 3 | 8 | 2 | 3 | 3 | 5 | 1 |  | ns | ns |
| 2 | 7 | 2 | 2 | 2 | 6 | 0 | 1 |  | ns |
| 1 | 7 | 2 | 2 | 1 | 6 | 1 | 0 | 0 |  |
